# Perceived factors associated with self-reported AI-assisted academic cheating among undergraduate students: Evidence from ethical ambiguity, technological affordances, institutional gaps, academic pressure, and peer comparison

**DOI:** 10.1371/journal.pone.0359549

**Published:** 2026-09-28

**Authors:** Hanh Van Nguyen, Mai Thi-Thuy Duong

**Affiliations:** Faculty of Education, Hanoi University of Science and Technology, Hanoi, Vietnam; Western Carolina University, UNITED STATES OF AMERICA

## Abstract

The rapid development of generative artificial intelligence (AI) has made academic cheating in higher education increasingly complex and difficult to regulate. Using a cross-sectional self-report survey of 863 undergraduate students at a university of science and technology in Vietnam, this study examined the latent structure of students’ perceptions of factors associated with AI-assisted academic cheating and the associations of the resulting constructs with self-reported cheating behaviors. Data were analyzed using exploratory factor analysis, confirmatory factor analysis, and covariance-based structural equation modeling. The results supported a three-factor structure consisting of AI-Assisted Academic Cheating Behaviors (AICB), Ethical Ambiguity, Technological Affordances, and Institutional Gaps (ETIG), and Academic Pressure and Peer Comparison (APPC). In the structural model, ETIG was positively associated with AICB (β = 0.228, p < 0.001), whereas APPC showed a small negative association (β = −0.148, p = 0.022). Gender was also positively associated with AICB (β = 0.142, p < 0.001), with male students tending to report higher AICB scores than female students. Student seniority was not significantly associated with AICB (β = 0.018, p = 0.622). Field of study was positively associated with AICB (β = 0.104, p = 0.008), with non-STEM students tending to report higher AICB scores than STEM students. The model accounted for approximately 5% of the variance in AICB, indicating modest explanatory power. Overall, the findings suggest that unclear boundaries around acceptable AI use, ease of access to AI tools and difficulty detecting AI-generated work, gaps in institutional guidance, and academic pressure and peer comparison may warrant further examination in relation to AI-assisted academic cheating.

## 1. Introduction

Academic cheating has long been a persistent concern in higher education [1], because it undermines the core values of academic work, including honesty, fairness, responsibility, and respect for intellectual effort [2]. Behaviors such as plagiarism, unauthorized use of materials during examinations, and fabrication of research data not only damage educational quality but also weaken trust in the authenticity of learning outcomes and the value of academic degrees [3,4]. Studies have shown that academic cheating is prevalent across many higher education systems and is often associated with institutional contexts, peer norms, and cultures of integrity [5,6]. Furthermore, the ways students understand and practice academic integrity are strongly shaped by academic culture, pedagogical practices, and broader social values, meaning that the definition of cheating is not always consistent across contexts [7]. However, the emergence of generative artificial intelligence (GenAI) has not alleviated this longstanding problem. Instead, GenAI has reshaped academic cheating in ways that are more complex and difficult to regulate in higher education [8]. The concept of AI-assisted academic cheating refers to the unethical use of AI technologies to produce academic work without genuine student effort [9], undermining fairness and devaluing educational credentials [10,11].

AI systems based on large language models such as ChatGPT are capable of generating coherent academic text, summarizing documents, supporting argumentation, and addressing a wide range of learning tasks [12–16]. These tools offer substantial opportunities for personalized learning and improved student learning efficiency [15,17]. AI tools such as ChatGPT have also demonstrated impressive performance by passing standardized tests and professional examinations in various fields such as law [18] or medicine [19,20]. At the same time, AI's powerful content generation capacity also increases the risk of misuse in academic assessment contexts [4]. When students can use AI to generate complete academic products within a very short time, the boundary between using AI as a legitimate learning support tool and using it to replace personal learning effort becomes increasingly blurred [3,4]. Moreover, AI-generated content is often difficult to detect through conventional plagiarism detection tools, making the control of academic cheating more complicated in contemporary higher education [21].

Unlike traditional forms of academic cheating, which are often easier to identify, AI-assisted cheating exists along a broad spectrum and frequently falls within gray areas between integrity and fraud [22]. Students may use AI to complete entire assignments, generate fake data or research results, paraphrase content to avoid plagiarism detection, or use AI in situations where instructors have explicitly prohibited it [22]. Therefore, AI-assisted cheating should not be understood as a single behavior, but as a spectrum of academic practices that may include full delegation of academic tasks to AI, submission of AI-generated work without intellectual contribution, lack of transparency in acknowledging AI assistance, and use of AI in violation of ethical or instructional guidelines [22]. This classification suggests that AI-related academic cheating exists across a wide spectrum, blurring the lines between authentic learning and outsourced academic work [8].

Beyond identifying forms of cheating behavior, previous research has also examined academic, ethical, technological, and institutional conditions associated with AI-assisted academic cheating. Academic pressure has frequently been associated with dishonest academic practices [23]. Heavy workloads, pressure to achieve high academic performance, and intense competition may be relevant to students’ inappropriate use of AI in academic work [23]. Ethical ambiguity may also be relevant when students are uncertain about the boundary between legitimate AI assistance and academic misconduct [4]. Technological characteristics such as ease of use and difficulty of detection may also be relevant to students’ perceptions of opportunities for inappropriate AI use [15,21]. Institutional policies, instructor guidance, and peer norms have also been identified as relevant contextual conditions [4,23]. When educational institutions do not provide clear policies on AI use in learning, or when instructors fail to offer specific guidance on how to use AI responsibly [4], students may interpret and apply academic norms in different ways [24]. A recent qualitative study identified four categories of factors frequently mentioned by students regarding AI-assisted academic dishonesty: academic pressure, ethical ambiguity, strong AI capabilities, and gaps in institutional academic policy [22]. Taken together, this literature suggests that AI-assisted academic cheating should be examined in relation to the broader academic, ethical, technological, and institutional context [22].

While a growing body of research has emerged on AI-assisted academic cheating, the majority of empirical studies focus on describing the prevalence of this behavior [8], measuring cheating intentions [25], or examining students’ perceptions of whether AI use constitutes academic cheating [24]. Studies have shown that students may conceal cheating behavior when asked directly [23,26], while others have explained cheating intention using behavioral models such as the Theory of Planned Behavior [25]. However, these approaches focus primarily on whether students cheat, with less attention to students’ perceptions of academic pressure, ethical ambiguity, AI capabilities, and institutional policy gaps in relation to inappropriate AI use. This leads to the first research question:

*Research question 1 (RQ1): How are the dimensions identified from students’ perceptions of academic pressure, ethical ambiguity, AI capabilities, and institutional policy gaps associated with their self-reported AI-assisted academic cheating behaviors?*

This study also examines the roles of gender and student seniority as relevant variables that may further clarify differences in academic cheating tendencies. In this regard, a study conducted in Vietnam found differences by gender and student seniority in tendencies toward AI-assisted academic cheating [23]. In related work, one study found that female students reported witnessing more cheating behaviors at school than male students, whereas male students may hold more tolerant views toward academically dishonest behavior than female students [27]. Another report indicated that male students were more likely than female students to engage in academic cheating in both face-to-face and online educational settings [28]. However, one study found no significant difference between males and females in academic cheating behavior, suggesting that the influence of gender may depend on the research context and the type of cheating behavior considered [29]. Similarly, student seniority has also been considered a factor that may influence academic cheating behavior. Female students in higher years tended to cheat more, whereas male students showed similar cheating rates across seniority levels [23].

*Research question 2 (RQ2): How are gender and student seniority associated with students’ self-reported AI-assisted academic cheating behaviors?*

## 2. Methods

### 2.1. Research design

This study employed a cross-sectional self-report survey design to examine the latent structure of items assessing students’ perceptions of factors associated with AI-assisted academic cheating and the associations of the resulting constructs with self-reported AI-assisted academic cheating behaviors (AICB). The analysis proceeded in two stages. First, exploratory factor analysis (EFA) was used to identify the underlying structure of the perception items without imposing predetermined dimensions. This exploratory approach was appropriate because the items were developed from prior qualitative findings rather than from an established measurement model. Second, covariance-based structural equation modeling (CB-SEM) was used to evaluate the measurement model of the constructs identified through EFA and to estimate their structural associations with AICB. Gender, student seniority, and field of study were also included as observed background variables in the structural model.

The questionnaire was administered anonymously online to reduce social pressure and encourage more honest responses to sensitive questions about academic cheating [26]. Before participation, students were informed about the purpose of the study, the anonymity of their responses, their voluntary participation, and their right to withdraw. Completion of the questionnaire was considered informed consent. The study was conducted in accordance with ethical guidelines and was approved by the Scientific Board of the Faculty of Education, Hanoi University of Science and Technology, Vietnam (FED-SRB-2025-010, date: October 1, 2025).

### 2.2. Participants

The participants were undergraduate students enrolled at a university of science and technology in Vietnam. Data were collected through an anonymous online survey distributed to students from a range of academic programs within the university. Convenience sampling was used because it was feasible to access student groups from different disciplines. The survey was conducted between October 25 and November 22, 2025. A total of 881 responses were received. After incomplete or invalid questionnaires had been removed, 863 responses were retained for subsequent statistical analyses. Table 1 presents the distribution of participants by gender, student seniority, and field of study.

**Table 1:** Participant distribution by gender, student seniority, and field of study.

Variable	Category	STEM n (%)	Non-STEM n (%)	Total n (%)
***Gender***	Female	152 (61.3)	96 (38.7)	248 (28.7)
	Male	550 (89.4)	65 (10.6)	615 (71.3)
***Student seniority***	First year	2 (2.6)	74 (97.4)	76 (8.8)
	Second year	420 (88.4)	55 (11.6)	475 (55.0)
	Third year	159 (89.8)	18 (10.2)	177 (20.5)
	Fourth year and above	121 (89.6)	14 (10.4)	135 (15.6)
	Total	702 (81.3)	161 (18.7)	863 (100.0)

As shown in Table 1, of the 863 students included in the final sample, most came from STEM (Science, Technology, Engineering, and Mathematics) fields (n = 702, 81.3%), while a smaller proportion came from non-STEM fields (n = 161, 18.7%). Regarding gender, male students accounted for 71.3% of the sample, whereas female students accounted for 28.7%. This distribution generally reflects the characteristics of technical universities, where male students often account for the majority in many engineering and technology programs in Vietnam [30–33]. Within the male group, 89.4% were from STEM fields and 10.6% were from non-STEM fields. Among female students, 61.3% were from STEM fields and 38.7% were from non-STEM fields. Thus, although both male and female students were concentrated in STEM fields, the proportion of non-STEM students was higher among female students than among male students.

The distribution by student seniority was also uneven. Second-year students formed the largest group, representing 55.0% of the sample, followed by third-year students (20.5%), fourth-year students and above (15.6%), and first-year students (8.8%). Most second-, third-, and fourth-year students were from STEM fields, whereas first-year students were predominantly from non-STEM fields. Overall, the sample was predominantly drawn from STEM programs, with field of study unevenly distributed across gender and student seniority groups. Therefore, field of study was included as an observed control variable in the structural model to account for potential confounding related to disciplinary background.

### 2.3. Survey instrument

Drawing on findings from a previous qualitative study of AI-assisted academic cheating in higher education [22], the survey was designed to assess students’ self-reported cheating behaviors, their perceptions of factors associated with such behaviors, and selected background characteristics. The AICB section initially comprised eleven items assessing students’ self-reported engagement in specific forms of inappropriate AI use in academic work. One conceptually overlapping item was removed before the final analyses, leaving ten items for EFA, CFA, and structural modeling. Participants indicated how often they had engaged in each behavior on a five-point frequency scale ranging from 1 (Never) to 5 (Very often). A further eleven items assessed students’ perceptions of factors potentially associated with AI-assisted academic cheating. These items were not assigned a priori to predefined subscales; instead, their empirical structure was examined using EFA. Responses were recorded on a five-point agreement scale ranging from 1 (Strongly disagree) to 5 (Strongly agree).

The questionnaire also collected information on gender, student seniority, and field of study. Programs in science, technology, engineering, and mathematics were classified as STEM, whereas programs outside these disciplinary areas were classified as non-STEM. Gender was coded as 0 = female and 1 = male. Student seniority was coded as 1 = first year, 2 = second year, 3 = third year, and 4 = fourth year or above. Field of study was coded as 0 = STEM and 1 = non-STEM.

### 2.4. Data analysis

The data were analyzed using SPSS version 26 and AMOS version 24. The significance level was set at 5%. Data analysis followed a sequential procedure including descriptive statistics, exploratory factor analysis, confirmatory factor analysis, and structural model testing. First, descriptive statistics were calculated for item responses. The normality of observed variables was examined using skewness and kurtosis. Skewness values within ±2 and kurtosis values within ±4 were considered acceptable [34].

Second, EFA was conducted on the 21 items retained for analysis to examine their underlying factor structure. The suitability of the data for EFA was evaluated using the Kaiser–Meyer–Olkin (KMO) measure and Bartlett’s test of sphericity. A KMO value of 0.70 or higher was considered acceptable, and a significant Bartlett’s test at p < 0.05 indicated that the correlation matrix was appropriate for factor analysis [35]. Factors were extracted using principal axis factoring because the purpose was to identify latent constructs. Because the factors were expected to be conceptually related, oblique rotation was used. Items were retained when they had a primary factor loading of at least 0.50, with 0.70 or higher considered preferable [35]. Items were also examined for cross-loadings, and a difference of at least 0.20 between the primary loading and any cross-loading was considered acceptable [36].

Third, confirmatory factor analysis (CFA) was conducted to validate the measurement model derived from EFA. Standardized factor loadings of 0.70 or higher were considered preferable, loadings from 0.60 to 0.69 were considered acceptable, and loadings of 0.50 to 0.59 were considered conditionally acceptable when theoretically justified [36]. Internal consistency was assessed using Cronbach’s alpha and composite reliability (CR). Cronbach’s alpha values of 0.70 or higher were considered preferable, while values between 0.60 and 0.70 were considered acceptable [36,37]. CR values of 0.70 or higher indicated adequate reliability, whereas values above 0.95 could suggest item redundancy [35]. Convergent validity was evaluated using average variance extracted (AVE), with values of 0.50 or higher indicating adequate convergence [35]. When the AVE was slightly below 0.50 (0.40 or higher), the construct was interpreted cautiously and retained only when CR was adequate and the construct was theoretically coherent, following the logic that adequate composite reliability may support convergent validity when AVE is marginal [38]. Discriminant validity was assessed using the Fornell–Larcker criterion and the heterotrait–monotrait ratio of correlations (HTMT). For the Fornell–Larcker criterion, discriminant validity was supported when the square root of AVE for each construct was greater than its correlations with other constructs [36]. For HTMT, values below 0.85 indicated strict discriminant validity, while values below 0.90 were considered acceptable for conceptually related constructs [35].

Finally, CB-SEM was used to examine associations between the identified latent constructs and self-reported AICB. Gender, student seniority, and field of study were entered as observed background variables. Model fit was evaluated using multiple indices. The chi-square/degrees of freedom ratio (χ²/df) below 3 indicated good fit, while values below 5 were considered acceptable [39]. CFI and TLI values of 0.90 or higher indicated acceptable fit, and values of 0.95 or higher indicated good fit [40,41]. RMSEA values of 0.08 or lower indicated acceptable fit, and values of 0.06 or lower indicated good fit [40,41]. SRMR values of 0.08 or lower were considered acceptable [40,41]. Structural paths were interpreted as statistically significant when p < 0.05. The explained variance of AI-assisted academic cheating behaviors was reported using R^2^.

## 3. Results

### 3.1. Descriptive statistics

Table 2 presents descriptive statistics for the survey items assessing students’ self-reported AI-assisted academic cheating behaviors and their perceptions of factors associated with such behaviors.

**Table 2:** Descriptive statistics for survey items included in the analyses.

Item code	Item label	M	SD	Skewness	Kurtosis
AICB1	Using AI to complete an entire assignment	2.242	1.084	0.429	−0.604
AICB2	Using AI to answer exam questions	2.204	1.090	0.392	−0.803
AICB3	Using AI to fabricate or manipulate research data or results	1.663	0.996	1.371	1.035
AICB4	Submitting AI-generated work as one’s own	2.053	1.130	0.702	−0.521
AICB5	Submitting AI-generated work with minimal intellectual contribution	2.061	1.088	0.697	−0.341
AICB6	Paraphrasing AI-generated content to avoid detection	2.423	1.164	0.251	−0.880
AICB7	Using AI-fabricated references and citations	1.926	1.136	0.916	−0.248
AICB8	Accepting AI outputs without critical evaluation	2.003	1.105	0.810	−0.242
AICB9	Using AI when the teacher has prohibited it	1.645	1.027	1.420	1.007
AICB10	Using AI to appropriate or rewrite others’ gray literature	1.662	1.028	1.400	1.024
PI1	Unclear boundaries between AI assistance and cheating	3.625	0.863	−0.562	0.525
PI2	Inadequate understanding of academic integrity in AI use	3.664	0.885	−0.691	0.658
PI3	Misbelief that AI-assisted cheating is harmless	3.659	0.891	−0.601	0.314
PI4	Academic performance pressure	3.811	0.908	−0.589	0.069
PI5	Overloaded academic schedules	3.846	0.859	−0.611	0.358
PI6	Social comparison with peers	3.508	0.947	−0.321	−0.343
PI7	Ease of AI use and difficulty of detection	3.645	0.922	−0.574	0.071
PI8	Overreliance on AI tools	3.648	0.897	−0.555	0.182
PI9	Absence of clear institutional AI policies	3.626	0.887	−0.554	0.392
PI10	Limited instructor guidance on ethical AI use	3.479	0.969	−0.373	−0.339
PI11	Lack of monitoring and detection mechanisms	3.552	0.945	−0.554	0.125

**Note.** AICB = AI-assisted cheating behavior; PI = item assessing students’ perceptions of factors associated with AI-assisted academic cheating; M = mean; SD = standard deviation. For AICB items, response options ranged from 1 = Never to 5 = Very often. For PI, response options ranged from 1 = Strongly disagree to 5 = Strongly agree.

As shown in Table 2, mean scores for the AICB items ranged from 1.645 to 2.423 and were generally below the midpoint of the five-point frequency scale, indicating relatively low levels of self-reported engagement in these behaviors. The items assessing students’ perceptions of factors associated with AI-assisted academic cheating showed higher mean scores, ranging from 3.479 to 3.846. Among the highest-scoring items were overloaded academic schedules (PI5, M = 3.846) and academic performance pressure (PI4, M = 3.811). Overall, the descriptive results show a contrast between relatively low levels of self-reported cheating behavior and comparatively high agreement with statements about factors perceived to be associated with AI-assisted academic cheating. In addition, all skewness values were within ±2 and all kurtosis values were within ±4, indicating that the item distributions satisfied the normality criteria.

### 3.2. Exploratory factor analysis

Table 3 reports the Kaiser–Meyer–Olkin measure and Bartlett’s test of sphericity used to assess whether the data were suitable for exploratory factor analysis.

**Table 3:** KMO and Bartlett’s test for exploratory factor analysis.

Indicator	Observed value	Interpretation
KMO	0.921	Excellent sampling adequacy
Bartlett’s test of sphericity, χ²	10119.069	
df	210	Degrees of freedom for Bartlett’s test
p	<0.001	The correlation matrix was factorable

**Note.** KMO = Kaiser–Meyer–Olkin measure of sampling adequacy. A significant Bartlett’s test of sphericity indicates that the correlation matrix is factorable and suitable for exploratory factor analysis.

The KMO value was 0.921, which was higher than the recommended threshold of 0.70. Bartlett’s test of sphericity was also statistically significant, χ² = 10119.069, df = 210, p < 0.001. These results indicate that the correlation matrix was factorable and that the dataset was appropriate for EFA.

Fig 1 and Table 4 summarize the evidence used to determine the number of retained factors, including the scree plot, eigenvalues, and explained variance.

**Table 4:** Total variance explained by the retained factors.

Factor	Initial eigenvalue	% of variance	Cumulative %	Extraction sums of squared loadings	Rotation sums of squared loadings
Factor 1	6.582	31.342	31.342	6.172	5.907
Factor 2	4.880	23.237	54.579	4.410	3.691
Factor 3	1.211	5.765	60.344	0.748	1.732

**Note.** Extraction method: principal axis factoring. The number of retained factors was determined based on the eigenvalues, the scree plot, theoretical interpretability, and the conceptual structure of the study.

**Fig 1:**
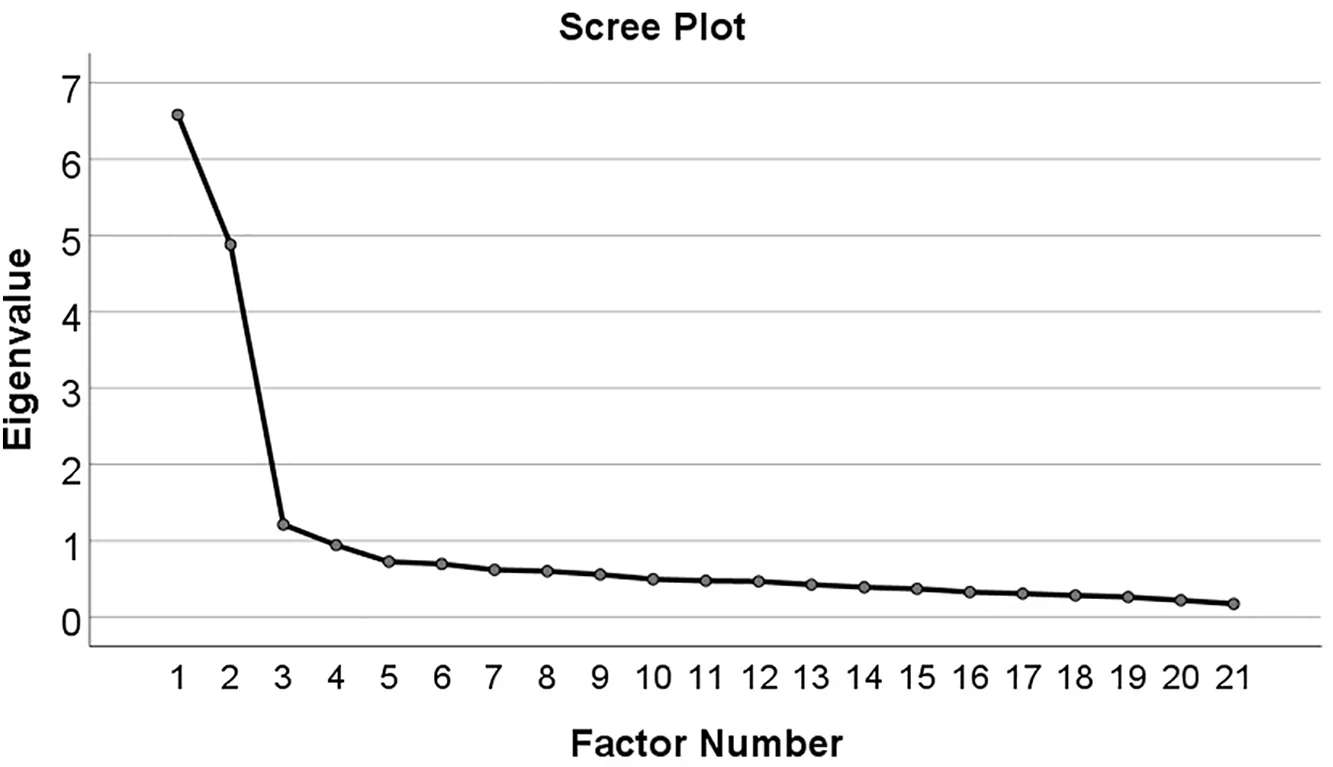
Scree plot of the exploratory factor analysis.

The scree plot, together with the eigenvalues and theoretical interpretability, supported a three-factor solution. The first three factors had eigenvalues greater than 1, suggesting that the analyzed item set could be represented by three broad latent constructs. Factor 1 accounted for 31.342% of the variance, Factor 2 accounted for 23.237%, and Factor 3 accounted for 5.765%. The cumulative explained variance of 60.344% indicated that the three-factor solution captured an acceptable proportion of the item variance. However, the distribution of explained variance also suggested that the first two factors were more dominant than the third factor.

Table 5 presents the rotated factor loadings from the exploratory factor analysis.

**Table 5:** Rotated factor loadings from exploratory factor analysis.

Item code	Factor 1	Factor 2	Factor 3	Decision
AICB1	**0.676**			Retained
AICB2	**0.637**			Retained
AICB3	**0.856**			Retained
AICB4	**0.759**			Retained
AICB5	**0.799**			Retained
AICB6	**0.643**			Retained
AICB7	**0.790**			Retained
AICB8	**0.775**			Retained
AICB9	**0.828**			Retained
AICB10	**0.851**			Retained
PI1		**0.575**		Retained
PI2		**0.587**		Retained
PI3		**0.587**		Retained
PI4		0.409	**0.718**	Retained
PI5		0.409	**0.678**	Retained
PI6		0.339	**0.561**	Retained
PI7		**0.606**	0.357	Retained
PI8		**0.564**	0.332	Retained
PI9		**0.744**		Retained
PI10		**0.660**		Retained
PI11		**0.706**		Retained

**Note.** AICB = AI-assisted cheating behavior; PI = item assessing students’ perceptions of factors associated with AI-assisted academic cheating. Extraction method: principal axis factoring. Rotation method: oblique rotation. Only factor loadings ≥ .30 are displayed for readability. Items were retained when they showed a theoretically meaningful primary loading and no substantial cross-loading. Factor labels were assigned after examining the retained items.

All ten AI-assisted academic cheating behavior items loaded on Factor 1, with loadings ranging from 0.637 to 0.856. These loadings met or exceeded the acceptable threshold of 0.50. Therefore, Factor 1 was labeled “AI-Assisted Academic Cheating Behaviors” (AICB). This factor represented students’ self-reported engagement in different forms of inappropriate AI use in academic work.

Factor 2 included PI1, PI2, PI3, PI7, PI8, PI9, PI10, and PI11. These items reflected unclear ethical boundaries, misunderstanding of academic integrity, ease of AI use and difficulty of detection, overreliance on AI tools, unclear institutional policies, limited instructor guidance, and weak monitoring mechanisms. The primary loadings ranged from 0.564 to 0.744, meeting the acceptable loading criterion. This factor was labeled “Ethical Ambiguity, Technological Affordances, and Institutional Gaps” (ETIG).

Factor 3 included PI4, PI5, and PI6, which referred to academic performance pressure, overloaded academic schedules, and social comparison with peers. PI4 and PI5 showed acceptable loadings, while PI6 had a weaker loading. Nevertheless, PI6 was retained because it was theoretically relevant to the pressure-related dimension and contributed to the conceptual meaning of the factor. This factor was labeled “Academic Pressure and Peer Comparison” (APPC).

Overall, the EFA results suggested that students’ self-reported AICB formed a distinct behavioral construct. The items assessing students’ perceptions of factors associated with AI-assisted academic cheating clustered into two broader dimensions: ETIG and APPC. This finding provided the empirical basis for the subsequent CFA and structural model testing.

### 3.3. Confirmatory factor analysis

Fig 2 presents the CFA model based on the three-factor structure identified through EFA. The CFA model included three latent constructs: AICB, ETIG, and APPC. This model was tested to examine whether the factor structure obtained from EFA provided an acceptable measurement model for the data.

**Fig 2:**
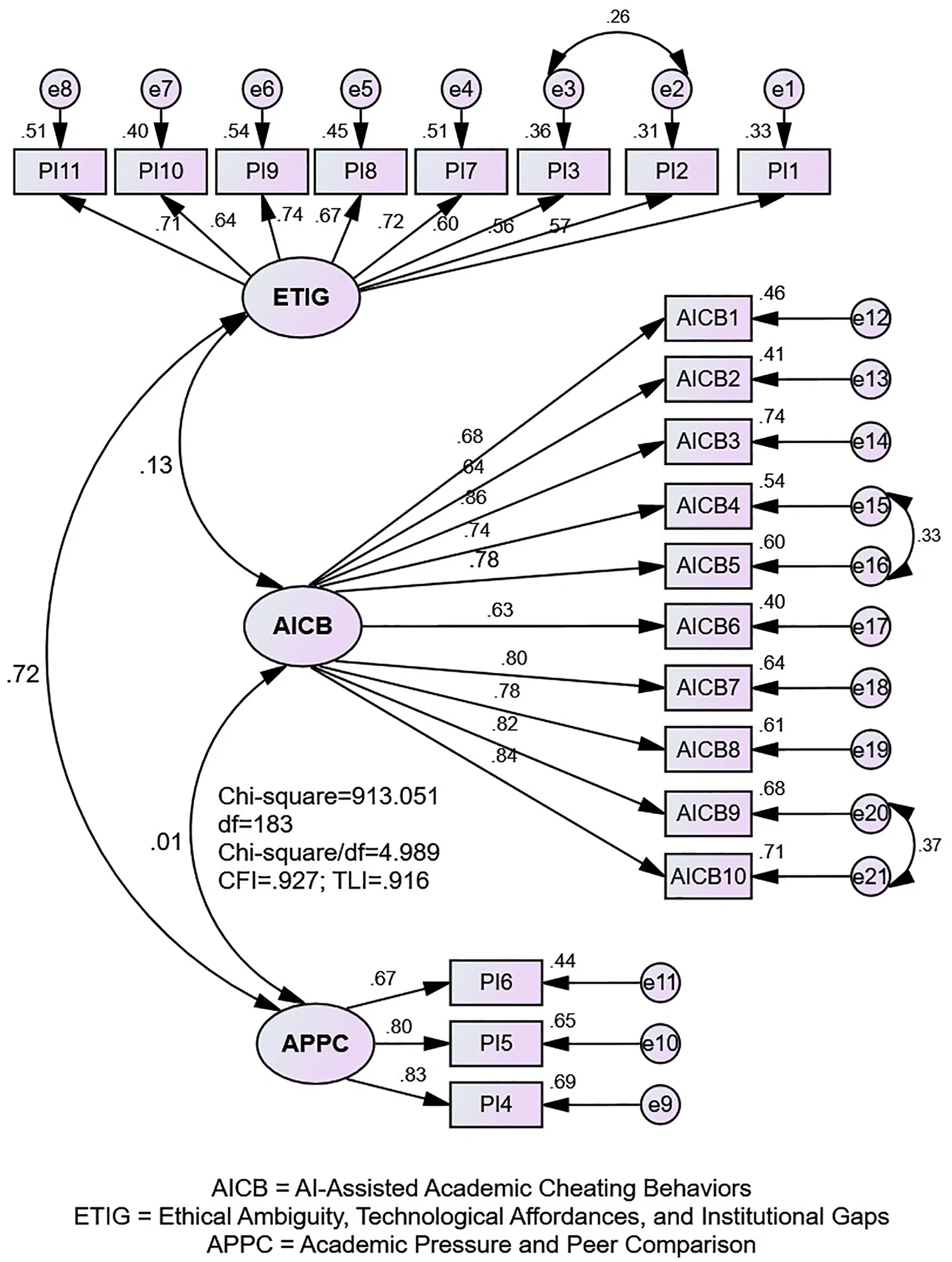
Confirmatory factor analysis model of the three-factor measurement structure.

Table 6 reports the standardized factor loadings, Cronbach’s alpha, composite reliability, and average variance extracted for the three latent constructs.

**Table 6:** Standardized factor loadings, reliability, and convergent validity.

Latent construct	Item code	λ	Cronbach’s α	CR	AVE
AI-Assisted Academic Cheating Behaviors (AICB)	AICB1	0.677	0.932	0.932	0.580
	AICB2	0.641			
	AICB3	0.858			
	AICB4	0.738			
	AICB5	0.776			
	AICB6	0.635			
	AICB7	0.799			
	AICB8	0.782			
	AICB9	0.822			
	AICB10	0.845			
Ethical Ambiguity, Technological Affordances, and Institutional Gaps (ETIG)	PI1	0.570	0.858	0.855	0.428
	PI2	0.560			
	PI3	0.603			
	PI7	0.716			
	PI8	0.671			
	PI9	0.738			
	PI10	0.636			
	PI11	0.712			
Academic Pressure and Peer Comparison (APPC)	PI4	0.832	0.807	0.813	0.594
	PI5	0.804			
	PI6	0.665			

**Note.** AICB = AI-assisted cheating behavior; PI = item assessing students’ perceptions of factors associated with AI-assisted academic cheating; λ = standardized factor loading; CR = composite reliability; AVE = average variance extracted. Standardized loadings are from the CFA measurement model. Cronbach’s α, CR, and AVE are reported at the construct level. The latent construct labels were assigned based on the EFA results and theoretical interpretability.

For AICB, standardized factor loadings ranged from 0.635 to 0.858. Although some loadings were below the preferable threshold of 0.70, all were above 0.60 and therefore remained within an acceptable range. The construct also showed strong internal consistency, with Cronbach’s α = 0.932 and CR = 0.932. Its AVE was 0.580, exceeding the 0.50 threshold for convergent validity.

For ETIG, standardized factor loadings ranged from 0.560 to 0.738. Several items had loadings between 0.50 and 0.60, which suggests that this construct should be interpreted with some caution. Its AVE was 0.428, which was below the recommended threshold of 0.50 but remained within the marginal range specified in the data analysis procedure. However, the construct was retained because its composite reliability was adequate (CR = 0.855), the standardized factor loadings were theoretically meaningful, and the construct was important for the structural model. This interpretation is consistent with the view that a construct with AVE below 0.50 may still be considered acceptable when composite reliability exceeds 0.70, although it should be interpreted cautiously.

For APPC, standardized factor loadings ranged from 0.665 to 0.832. These values were acceptable, with two of the three items exceeding 0.80. The construct also showed adequate reliability, with Cronbach’s α = 0.807 and CR = 0.813. Its AVE was 0.594, exceeding the recommended threshold of 0.50. Thus, this construct showed acceptable reliability and convergent validity.

Table 7 presents the model fit indices for the measurement model.

**Table 7:** Model fit indices for the measurement model.

Model	χ²	df	χ²/df	CFI	TLI	RMSEA	90% CI for RMSEA	SRMR
Overall measurement model	913.051	183	4.989	0.927	0.916	0.068	0.064–0.072	0.0572

**Note.** χ² = chi-square; df = degrees of freedom; CFI = comparative fit index; TLI = Tucker–Lewis index; RMSEA = root mean square error of approximation; SRMR = standardized root mean square residual.

The CFA model showed acceptable overall fit to the data: χ²(183) = 913.051, χ²/df = 4.989, CFI = 0.927, TLI = 0.916, RMSEA = 0.068 (90% CI [0.064, 0.072]), and SRMR = 0.0572. The χ²/df value was below 5, indicating acceptable fit. The CFI and TLI values were above 0.90, indicating acceptable incremental fit. The RMSEA and SRMR values were both below 0.08, indicating acceptable approximate and residual fit. Overall, the model fit indices suggested that the three-factor measurement model was acceptable.

Table 8 reports the latent construct correlations and the Fornell–Larcker criterion for discriminant validity.

**Table 8:** Latent construct correlations and discriminant validity.

Construct	AICB	ETIG	APPC
**AICB**	**0.761**		
**ETIG**	0.125	**0.654**	
**APPC**	0.014	0.716	**0.770**

**Note.** AICB = AI-Assisted Academic Cheating Behaviors; ETIG = Ethical Ambiguity, Technological Affordances, and Institutional Gaps; APPC = Academic Pressure and Peer Comparison. Diagonal values represent the square root of AVE. Off-diagonal values represent latent construct correlations. Discriminant validity is supported when the square root of AVE for each construct is greater than its correlations with other constructs.

As shown in Table 8, the Fornell–Larcker criterion was satisfied for AICB relative to the other constructs but was not fully satisfied for the ETIG–APPC pair. The square root of AVE for ETIG (0.654) was lower than its correlation with APPC (r = 0.716), indicating a potential concern regarding discriminant validity between these two constructs. Given this result, discriminant validity was further examined using the HTMT ratio.

Table 9 presents the HTMT ratios among the latent constructs.

**Table 9:** HTMT ratios among latent constructs.

Construct pair	HTMT
**ETIG – APPC**	0.719
**ETIG – AICB**	0.160
**APPC – AICB**	0.138

**Note.** HTMT = heterotrait–monotrait ratio of correlations. Values below  .85 indicate strict discriminant validity, whereas values below  .90 may be acceptable for conceptually related constructs.

As shown in Table 9, all HTMT values were below 0.85, including the ETIG–APPC value of 0.719, providing support for discriminant validity according to the HTMT criterion. Although the Fornell–Larcker criterion was not met for ETIG and APPC, their separation was retained because the HTMT value (0.719) was below 0.85, the EFA produced two conceptually distinct item clusters, and both constructs showed adequate composite reliability. These complementary results supported treating ETIG and APPC as separate constructs in the structural model.

### 3.4. Structural model testing

Fig 3 presents the structural model used to examine the associations of ETIG and APPC with self-reported AICB. Gender, student seniority, and field of study were included as observed background variables.

**Fig 3:**
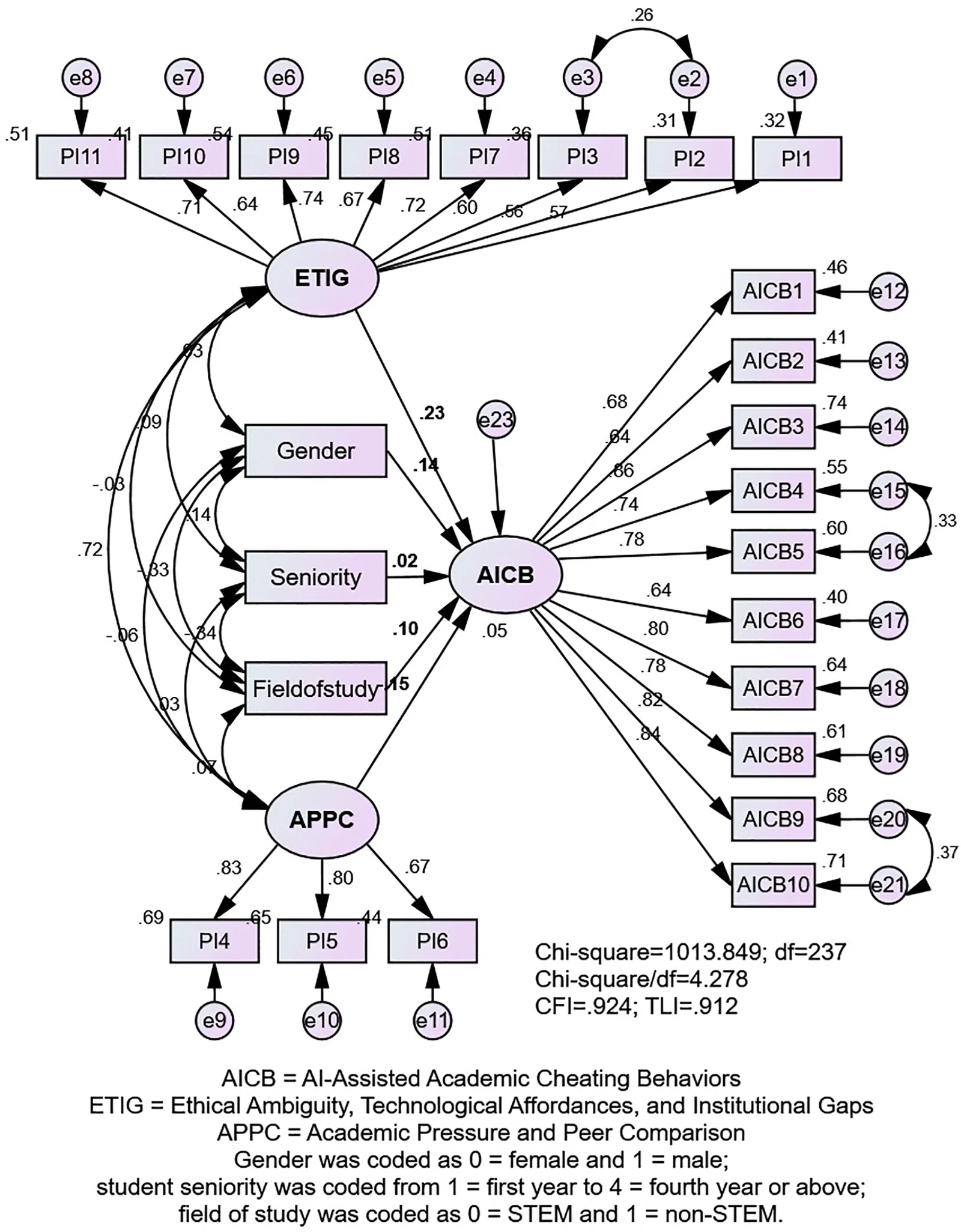
Structural model of associations between the identified dimensions, background variables, and self-reported AI-assisted academic cheating behaviors.

Table 10 presents the fit indices for the structural model.

**Table 10:** Model fit indices for the structural model.

Model	χ²	df	χ²/df	CFI	TLI	RMSEA	90% CI for RMSEA	SRMR
Structural model	1013.849	237	4.278	0.924	0.912	0.062	0.058–0.066	0.0523

**Note.** χ² = chi-square; df = degrees of freedom; CFI = comparative fit index; TLI = Tucker–Lewis index; RMSEA = root mean square error of approximation; SRMR = standardized root mean square residual. The structural model was evaluated after the overall CFA measurement model had been established.

As shown in Table 10, the structural model showed acceptable fit to the data, with χ²(237) = 1013.849, χ²/df = 4.278, CFI = 0.924, TLI = 0.912, RMSEA = 0.062 (90% CI [0.058, 0.066]), and SRMR = 0.0523. Overall, these values met the prespecified criteria for acceptable model fit.

Table 11 reports the standardized path coefficients for the structural model.

**Table 11:** Structural path estimates for associations with self-reported AI-assisted academic cheating behaviors.

Path	β	SE	CR	p
ETIG → AICB	0.228	0.070	3.555	<0.001
APPC → AICB	−0.148	0.075	−2.297	0.022
Gender → AICB	0.142	0.061	3.791	<0.001
Student seniority → AICB	0.018	0.032	0.493	0.622
Field of study → AICB	0.104	0.073	2.654	0.008

**Note.** AICB = AI-Assisted Academic Cheating Behaviors; ETIG = Ethical Ambiguity, Technological Affordances, and Institutional Gaps; APPC = Academic Pressure and Peer Comparison; β = standardized path coefficient; SE = standard error; CR = critical ratio. SE and CR are based on the corresponding unstandardized path estimate. Gender was coded as 0 = female and 1 = male; field of study was coded as 0 = STEM and 1 = non-STEM. The structural model explained 5% of the variance in AICB (R² = 0.05).

As shown in Table 11, ETIG was positively associated with AICB (β = 0.228, SE = 0.070, CR = 3.555, p < 0.001), whereas APPC showed a small negative association (β = −0.148, SE = 0.075, CR = −2.297, p = 0.022). Among the observed background variables, gender was positively associated with AICB (β = 0.142, SE = 0.061, CR = 3.791, p < 0.001), indicating that male students tended to report higher AICB scores than female students. Field of study, which was included as a control variable, was also positively associated with AICB (β = 0.104, SE = 0.073, CR = 2.654, p = 0.008), with non-STEM students tending to report higher AICB scores than STEM students. For comparison, in an identical model that excluded field of study, the Gender → AICB association was β = .183 (p = .002). After field of study was included as a control variable, the coefficient decreased to β = .142 (p < .001), indicating that the gender association was modestly attenuated but remained statistically significant. In contrast, student seniority was not associated with AICB (β = 0.018, SE = 0.032, CR = 0.493, p = 0.622). Overall, the structural model accounted for approximately 5% of the variance in AICB. Although several statistically significant associations were identified, the model had modest explanatory power, and these findings should not be interpreted as evidence of causal relationships.

## 4. Discussion and Implications

### 4.1. Discussion

This study examined how students’ perceptions of academic pressure, ethical ambiguity, AI capabilities, and institutional policy gaps were empirically organized and how the resulting dimensions were associated with self-reported AICB. The analyses identified two dimensions, ETIG and APPC, which showed different patterns of association with AICB. The structural model also indicated associations involving gender and field of study, whereas student seniority was not significantly associated with AICB. Given the modest proportion of variance explained by the model, these findings should be interpreted as evidence of association rather than as strong explanatory or causal evidence.

In relation to RQ1, ETIG was positively associated with students’ self-reported AICB (β = 0.228, p < 0.001). Higher ETIG scores, reflecting unclear ethical boundaries, easy access to AI tools, difficulty detecting AI-assisted work, and gaps in institutional guidance, were associated with higher self-reported AICB. This pattern is consistent with previous research highlighting the difficulty of distinguishing legitimate AI assistance from academic misconduct and the challenges that generative AI creates for academic integrity policies and practices [3,4,21]. Importantly, the EFA showed that items concerning ethical ambiguity, technological affordances, and institutional policy gaps clustered within a single dimension rather than remaining separate. This finding suggests that students may perceive these issues as closely interconnected. For example, uncertainty about acceptable AI use may coexist with perceptions that AI-generated work is difficult to detect or that institutional and instructor guidance is insufficient. Therefore, the present findings support considering these ethical, technological, and institutional issues together when examining students’ self-reported AI-assisted academic cheating [22].

APPC showed a small negative association with AICB in the structural model (β = −0.148, p = 0.022), but this result requires particularly cautious interpretation. At the latent correlation level, APPC was essentially unrelated to AICB (r = 0.014), while APPC and ETIG were substantially correlated (r = 0.716). The negative structural coefficient represents the association between APPC and AICB after accounting for ETIG and the other variables in the model, rather than a simple inverse relationship between academic pressure and cheating. Therefore, it should not be interpreted as evidence that academic pressure or peer comparison reduces AI-assisted academic cheating. Academic pressure has been discussed in previous research as relevant to dishonest academic practices [23], and students in the present study also reported relatively high agreement with items concerning academic performance pressure and overloaded schedules. Taken together, these results suggest that the relationship between academic pressure, peer comparison, and AI-assisted cheating may be more complex than a straightforward positive or negative association and warrants further investigation.

In relation to RQ2, gender was positively associated with self-reported AICB (β = 0.142, p < 0.001), with male students tending to report higher AICB scores than female students. This pattern is broadly consistent with studies reporting higher levels of academic cheating or greater tolerance of dishonest academic behavior among male students [27,28], although previous findings have not been consistent across contexts [29]. The association observed in the present study was modest and should be interpreted cautiously because the sample was drawn from a single university and was unevenly distributed across gender, field of study, and student seniority. Although field of study was included as a control variable in the structural model, statistical adjustment cannot fully address these sample imbalances. Field of study was also positively associated with AICB (β = 0.104, p = 0.008), with non-STEM students tending to report higher AICB scores than STEM students. Because field of study was introduced primarily as a background control variable rather than as a focal variable in the research questions, this finding should be treated as supplementary. It should also be interpreted cautiously because STEM students constituted 81.3% of the sample and the distribution of field of study varied substantially across gender and student seniority groups. Further research with more balanced disciplinary samples is needed before drawing broader conclusions about differences between STEM and non-STEM students.

Student seniority was not significantly associated with AICB (β = 0.018, p = 0.622). This differs from previous findings in the Vietnamese context indicating variation in AI-assisted academic cheating across year levels [23]. The present result suggests that such differences may not be consistent across samples or institutional settings. Future studies could examine whether year-level differences depend on factors such as assessment practices, AI experience, or exposure to institutional guidance.

Overall, the structural model accounted for approximately 5% of the variance in students’ self-reported AICB, indicating that the observed associations represent only a limited part of a more complex phenomenon. Other potentially relevant variables, including perceived risk of detection, attitudes toward AI use, AI literacy, assessment design, moral disengagement, peer norms, and trust in institutional policies, were not examined in the present model. Future research should investigate these variables using broader samples and longitudinal or experimental designs that can provide stronger evidence about temporal and potentially causal relationships.

### 4.2. Practical implications

The findings point to several areas that higher education institutions may consider when developing guidance for student use of generative AI. The positive association between ETIG and self-reported AICB highlights the relevance of clear boundaries around acceptable AI use, explicit course-level expectations, and consistent institutional guidance. Students may benefit from concrete explanations of which uses of AI are permitted, which require disclosure, and which may constitute academic misconduct.

The small negative association between APPC and AICB should not be interpreted as evidence that academic pressure protects against cheating. Instead, the result indicates that academic pressure and peer comparison have a more complex relationship with AICB when considered alongside ETIG and the background variables. Therefore, institutions should avoid drawing intervention conclusions from this coefficient alone.

Gender and field of study were also associated with self-reported AICB, with male students tending to report higher scores than female students and non-STEM students tending to report higher scores than STEM students. However, both associations were modest and were observed in an unevenly distributed sample from a single institution. These findings may not provide a basis for targeting particular gender or disciplinary groups. Guidelines on AI use and academic integrity education should be provided across programs and academic years.

### 4.3. Limitations and future research directions

This study has several limitations that also point to directions for future research. First, the data were collected through convenience sampling from undergraduate students at a single university of science and technology in Vietnam. The sample was also unevenly distributed across gender, field of study, and student seniority. Therefore, future studies should examine the factor structure and structural associations in more diverse, multi-institutional samples and across different disciplinary contexts.

Second, the study relied on self-reported data about academically sensitive behaviors. Although participation was anonymous, underreporting remains possible. Several AICB items also showed low mean scores, which may have reduced the magnitude of the observed associations. Future research could use complementary approaches such as indirect questioning, list experiments, behavioral indicators, or mixed-methods designs to reduce reliance on direct self-report.

Third, the cross-sectional design does not establish temporal ordering or causal relationships among the variables. Longitudinal studies could clarify how students’ perceptions and self-reported AI-assisted cheating behaviors change over time, while appropriately designed experimental studies could provide stronger evidence regarding potential causal mechanisms.

Finally, the structural model accounted for only approximately 5% of the variance in self-reported AICB, indicating that substantial variation remains unexplained. Future studies should examine additional variables such as perceived risk of detection, attitudes toward AI use, AI literacy, assessment design, moral disengagement, peer norms, and trust in institutional policies.
