# Supplementary material for: Perceived factors associated with self-reported AI-assisted academic cheating among undergraduate students: Evidence from ethical ambiguity, technological affordances, institutional gaps, academic pressure, and peer comparison
Source: PLoS One. 2026 Sep 28;21(9):e0359549. doi: 10.1371/journal.pone.0359549 (PMC13618900; doi:10.1371/journal.pone.0359549)
Supplement: Supplementary file 2 [file pone.0359549.s004.pdf]

RESPONSE TO EDITOR AND REVIEWERS

Dear **Academic Editor and Reviewers,**

We sincerely thank the Academic Editor, Reviewer 1, and the editorial team for their careful re-evaluation of our manuscript and for the opportunity to revise it again. We have addressed the remaining concerns point by point. In this revision, we incorporated field of study into the structural model as an observed control variable and re-estimated the CB-SEM model; systematically revised the title, abstract, introduction, discussion, and implications to remove causal framing; and fully aligned Table 11 with the research-question framework. We also updated the affected Methods and Results sections, figures, tables, limitations, and references. All revisions are shown in the marked manuscript using Track Changes.

Response Academic Editor:

Thank you for submitting your manuscript to PLOS One. After careful consideration, we feel that it has merit but does not fully meet PLOS One's publication criteria as it currently stands. Therefore, we invite you to submit a revised version of the manuscript that addresses the points raised during the review process.

Response:

Thank you for the opportunity to submit a further revision. We have carefully addressed each of the remaining required revisions and Reviewer 1's concerns. The principal changes are: inclusion of field of study as an observed control variable in the structural model; re-estimation and full reporting of the revised CB-SEM results; systematic removal of causal wording throughout the manuscript; and revision of Table 11 so that it reports structural associations rather than hypothesis testing. The revised manuscript also updates all dependent sections so that the Methods, Results, Discussion, practical implications, and limitations are internally consistent. Detailed responses to each point are provided below.

Required Revisions

Please address the following items in your next revision:

Control for field of study. Reporting the gender distribution across STEM and non-STEM fields does not rule out confounding. You must incorporate appropriate statistical control and report how the gender–AICB association changes after adjustment.

Response:

We agree that descriptive reporting of STEM/non-STEM composition is not sufficient to address potential confounding. Field of study is now included directly in the structural model as an observed control variable, coded 0 = STEM and 1 = non-STEM. Gender was coded 0 = female and 1 = male, and student seniority was retained as an observed background variable. The coding and role of field of study are now described in Sections 2.2-2.4, and the revised model is reported in Section 3.4, Fig. 3, Table 10, and Table 11. The revised structural model showed acceptable fit, chi-square (237) = 1013.849, chi-square/df = 4.278, CFI = .924, TLI = .912, RMSEA = .062 (90% CI [.058, .066]), and SRMR = .0523. Field of study was positively associated with AICB (beta = .104, p = .008); given the coding, non-STEM students tended to report higher AICB scores than STEM students. The Gender -> AICB coefficient was beta = .183 (p = .002) in the model reported before field of study was introduced and beta = .142 (p < .001) in the current model including field of study. Thus, the gender association was not attenuated after field of study was included; it remained statistically significant and modest in magnitude. We have nevertheless tempered the interpretation because the sample remains unevenly distributed across gender and disciplinary groups. The revised interpretation is reflected in the Abstract, Results, Discussion (especially RQ2), practical implications, and limitations.

Temper causal language. Despite acknowledging the cross-sectional design, the manuscript still contains phrasing that implies causality (e.g., in the title, discussion, and implications). Revise the language throughout to reflect association rather than causation.

Response:

We agree and have systematically revised the manuscript to ensure that the study is framed in terms of association rather than causation. Most visibly, the title has been changed from the earlier causal formulation using 'Enablers' to: *'Perceived factors associated with self-reported AI-assisted academic cheating among undergraduate students: Evidence from*

ethical ambiguity, technological affordances, institutional gaps, academic pressure, and peer comparison' (p. 1). Across the Abstract, Introduction, Methods, Results, Discussion, and practical implications, study-specific terms such as 'driving factors,' 'enablers,' 'direct effects,' and other causal formulations were replaced with language such as 'associated with,' 'relationship,' and 'factors associated with.' The Discussion now explicitly states that the findings are evidence of association rather than strong explanatory or causal evidence. We also added a specific caution regarding the negative APPC coefficient: it is not interpreted as evidence that academic pressure or peer comparison reduces cheating. The practical implications are now framed as areas institutions may consider, rather than as interventions demonstrated to reduce AICB. Finally, the limitations explicitly state that the cross-sectional design does not establish temporal ordering or causal relationships.

Align Table 11 terminology. Your revised framework uses research questions, yet Table 11 retains hypothesis-testing terminology such as "hypothesis testing" and "Supported/Not supported." Revise this table to be consistent with your exploratory research question framework.

Response:

Thank you. Table 11 has been fully revised to align with the research-question framework. Its title is now *'Structural path estimates for associations with self-reported AI-assisted academic cheating behaviors'*. The former 'Result' column and the labels 'Supported/Not supported' have been removed, as has the note defining when a hypothesis is supported. The table now reports only the path, standardized coefficient (beta), standard error (SE), critical ratio (CR), and p value. The accompanying text interprets these estimates as statistical associations. This terminology is now consistent with RQ1 and RQ2 and with the exploratory framing used throughout the manuscript.

Your revisions thus far have partially addressed these points, but the remaining issues listed above must be conclusively resolved. I require a clear, point-by-point explanation of how

the current revision satisfies each of these original requirements and the reviewer's new concerns, so that no outstanding issues remain

Response:

We appreciate this instruction and have structured the present rebuttal accordingly. Each remaining issue is addressed separately with (i) the specific revision made, (ii) the relevant quantitative result where applicable, and (iii) the location of the change in the revised manuscript. In particular, the field-of-study concern is resolved through direct statistical control and explicit reporting of the Gender -> AICB coefficient before and after inclusion of field of study; causal language has been revised throughout the manuscript rather than only in isolated sections; and Table 11 has been fully converted from hypothesis-testing terminology to association-based reporting. The corresponding responses to Reviewer 1 below provide the same point-by-point clarification so that the editor and reviewer can readily verify each revision.

Response to Journal Requirements:

If the reviewer comments include a recommendation to cite specific previously published works, please review and evaluate these publications to determine whether they are relevant and should be cited. There is no requirement to cite these works unless the editor has indicated otherwise.

Response:

No reviewer in this round requested that specific publications be cited. We therefore did not add citations solely in response to a citation request. We re-evaluated the literature used to support the revised framing and retained citations only where they were relevant to the claims made in the manuscript.

Please review your reference list to ensure that it is complete and correct. If you have cited papers that have been retracted, please include the rationale for doing so in the manuscript text, or remove these references and replace them with relevant current references. Any changes to the reference list should be mentioned in the rebuttal letter that accompanies your

revised manuscript. If you need to cite a retracted article, indicate the article's retracted status in the References list and also include a citation and full reference for the retraction notice.

Response:

We reviewed the reference list for completeness, bibliographic accuracy, and retraction status. Several entries were corrected or updated during revision, including the DOI and publication details for the Fornell-Larcker reference, the DOI for the Narra Journal article, updated final publication information for the Journal of Applied Research in Higher Education article, and the DOI for the DASS-21 reference. We also checked the cited literature for retraction notices and did not identify a retracted publication among the references retained in the manuscript. No retracted article is cited as evidence in the revised manuscript.

Response to Reviewer 1

This study examines perceived factors associated with AI-assisted academic cheating among undergraduate students. The topic is timely and relevant, and the large sample is a clear strength. Compared with the original submission, the revised manuscript has improved considerably, particularly in the clarity with which the factor structure, sample characteristics, and study limitations are reported. However, several aspects of the interpretation and reporting of the findings require further clarification and revision.

Response:

Thank you for recognizing the improvements in the revised manuscript and for identifying the remaining issues requiring clarification. We have addressed each of the three concerns in the current revision. In particular, we have controlled for field of study in the structural model, further removed causal wording across the manuscript, and revised Table 11 to align completely with the research-question framework. Our detailed responses are provided below.

1. The revised manuscript now reports the gender distribution across STEM and non-STEM fields, which improves the transparency of the sample. However, this descriptive

information does not rule out potential confounding by field of study. The authors should adjust for field of study in the structural model or provide a sensitivity analysis stratified by STEM versus non-STEM status, and report whether the association between gender and AICB changes meaningfully after adjustment. If the association is substantially attenuated or becomes unstable, the interpretation of the gender finding should be tempered accordingly.

Response:

We agree. Field of study is now included as an observed control variable in the structural model (0 = STEM, 1 = non-STEM), rather than being treated only descriptively. The revised model is presented in Fig. 3 and Tables 10-11, and the relevant coding and analytical treatment are described in Sections 2.2-2.4. The model including field of study showed acceptable fit: chi-square(237) = 1013.849, chi-square/df = 4.278, CFI = .924, TLI = .912, RMSEA = .062 (90% CI [.058, .066]), and SRMR = .0523. Field of study was associated with AICB (beta = .104, p = .008), with non-STEM students tending to report higher AICB scores than STEM students. Importantly, the Gender -> AICB association was beta = .183 (p = .002) in the model reported before field of study was introduced and beta = .142 (p < .001) in the current model including field of study. Therefore, the gender association remained statistically significant and was not attenuated. We have nevertheless interpreted this association cautiously because the sample was unevenly distributed by gender and field of study and came from a single institution. This caution is now explicit in the Discussion and Limitations.

2. The revised manuscript appropriately acknowledges that the cross-sectional design does not support causal inference. However, relatively strong causal language remains in the title, discussion, and practical implications. Given the cross-sectional, self-report design, the authors should further temper this wording to avoid implying causality.

Response:

We agree and have further tempered the language throughout the manuscript. The title no longer uses 'Enablers' and now explicitly uses 'factors associated with.' Study-specific causal terms such as 'driving factors,' 'direct effects,' and causal descriptions of the findings were

replaced with association-based wording. The Abstract and Results report statistical associations; the Discussion explicitly states that the findings should not be interpreted as causal evidence; and the practical implications are framed as areas for institutional attention rather than demonstrated intervention effects. We also explicitly state that the negative APPC coefficient should not be interpreted as evidence that academic pressure protects against or reduces AI-assisted cheating. The cross-sectional limitation is reiterated in Section 4.3.

3. The terminology in Table 11 is inconsistent with the revised research framework. The revised manuscript is now structured around research questions, whereas Table 11 still uses hypothesis-testing terms such as “hypothesis testing” and “Supported/Not supported.” Please revise the terminology in Table 11 to ensure consistency with the research questions and the exploratory framing of the revised manuscript.

Response:

Thank you. Table 11 has been revised to remove all hypothesis-testing terminology. It is now titled *'Structural path estimates for associations with self-reported AI-assisted academic cheating behaviors.'* The 'Supported/Not supported' judgments and the hypothesis-support note have been removed. The table now reports Path, beta, SE, CR, and p only, and the surrounding text discusses the coefficients as associations relevant to RQ1 and RQ2. This revision makes Table 11 fully consistent with the exploratory research-question framework.

Thank you for your time and consideration of our revised manuscript. We look forward to hearing from you.

Sincerely,

Hanh Van Nguyen
Faculty of Education, Hanoi University of Science and Technology, Hanoi, Vietnam
Email: hanh.nguyenvan@hust.edu.vn
