# Supplementary material for: Perceived factors associated with self-reported AI-assisted academic cheating among undergraduate students: Evidence from ethical ambiguity, technological affordances, institutional gaps, academic pressure, and peer comparison
Source: PLoS One. 2026 Sep 28;21(9):e0359549. doi: 10.1371/journal.pone.0359549 (PMC13618900; doi:10.1371/journal.pone.0359549)
Supplement: Supplementary file 3 [file pone.0359549.s001.docx]

This study focuses on the drivers of AI-assisted academic cheating among undergraduate students in the context of generative AI. The topic is practically relevant and has clear real-world value. The sample size is relatively large (N = 863), and the dataset has potential research value. However, the following issues remain:

1.The study’s target population is undergraduates, yet the four-category classification of cheating behaviors used in the paper is derived from qualitative findings from a postgraduate population (“AI-assisted academic cheating: a conceptual model based on postgraduate student voices,” DOI: 10.3389/fcomp.2025.1682190). The paper does not provide sufficient evidence that this four-dimensional structure shows clear empirical discriminant validity in an undergraduate sample. As indicated by the HTMT results in Table 6—AIContentSub–NonDisclosureAI = 0.966, AITaskDelegation–AIPolicyViolation = 0.926, AITaskDelegation–NonDisclosureAI = 0.917, and AIContentSub–AITaskDelegation = 0.905—HTMT values above 0.90 suggest these constructs overlap substantially in the undergraduate population and may effectively reflect the same concept, making it difficult to distinguish them clearly. It is recommended that highly redundant constructs be merged.

2.The study splits academic cheating into four distinct dimensions, but the theoretical framework does not offer differentiated mechanisms to explain why specific drivers would predict one type of cheating rather than another. All hypotheses are drawn from the findings of a single prior article, which results in 16 specific hypotheses that lack theoretical depth and amount to largely ad hoc variable testing. Moreover, as noted above, the cited article examines postgraduate students; applying its results directly to undergraduates without any justification is methodologically imprecise and difficult to defend.

3.In the limitations section, the authors acknowledge that the model has low explanatory power, yet the paper still uses wording such as “prominent role,” which does not align with the statistical results. The explained variance for cheating behaviors is R² = 0.034–0.055, accounting for only about 3%–6% of the variation. According to Cohen’s f² benchmarks, aside from PolicyGaps → AITaskDelegation (f² = 0.022), the effect sizes of the other significant paths are all below the threshold for a small effect, indicating extremely weak effects. It is recommended that the wording be revised to characterize the findings as “statistically significant but small in effect,” avoiding phrasing that could imply a strong influence.

4.The observed gender effects may be confounded by disciplinary/major-related variables. In the sample, 71.3% are male, and they are mainly from engineering/science fields, whereas most female participants are concentrated in humanities, education, social sciences, and related majors. The authors report a significant effect of gender on cheating behaviors but do not control for discipline/major in the structural model. Consider testing gender effects within disciplinary strata (i.e., stratifying by major/field) before reporting an association between gender and cheating; only if the effect remains significant should it be reported as related to cheating behavior.

5.The authors list social desirability bias as a limitation but do not further discuss its potential impact on the structural model estimates. Because academic cheating is a morally sensitive behavior, students may underreport or be reluctant to admit such behaviors, and many participants selected “never” for the relevant items. Restricted variance can attenuate estimated path coefficients, thereby reducing the model’s statistical power to detect potential relationships. The authors are encouraged to elaborate in the discussion on how this issue might affect path significance and effect-size estimation.

6.The current structural model is highly saturated, with four driving factors and two demographic variables simultaneously predicting four cheating dimensions, resulting in 24 hypothesized paths. This design resembles an all-inclusive enumeration model rather than a parsimonious theory-driven structural model. The authors should justify the current path specification more explicitly and consider simplifying the model after re-specifying the endogenous constructs.

7.The manuscript applies PLS-SEM to test a conceptual model, yet the rationale for choosing PLS-SEM is not sufficiently explained. Given the relatively large sample size (N = 863), the explicit hypotheses, and the confirmatory nature of the study, the authors should justify why PLS-SEM was preferred over covariance-based SEM approaches more commonly used for theory testing.
