# Supplementary material for: Perceived factors associated with self-reported AI-assisted academic cheating among undergraduate students: Evidence from ethical ambiguity, technological affordances, institutional gaps, academic pressure, and peer comparison
Source: PLoS One. 2026 Sep 28;21(9):e0359549. doi: 10.1371/journal.pone.0359549 (PMC13618900; doi:10.1371/journal.pone.0359549)
Supplement: Peer review history

# Peer review: Decision Letter 0

**DOI:** 10.1371/journal.pone.0359549.r001

**Authors:** Haiyang Chen

28 Apr 2026

Dear Dr. Van-Hanh,

Thank you for submitting your manuscript to PLOS ONE. After careful consideration, we feel that it has merit but does not fully meet PLOS ONE’s publication criteria as it currently stands. Therefore, we invite you to submit a revised version of the manuscript that addresses the points raised during the review process.

==============================

**ACADEMIC EDITOR:** plosone@plos.org

- A letter that responds to each point raised by the academic editor and reviewer(s). You should upload this letter as a separate file labeled 'Response to Reviewers'.
- A marked-up copy of your manuscript that highlights changes made to the original version. You should upload this as a separate file labeled 'Revised Manuscript with Track Changes'.
- An unmarked version of your revised paper without tracked changes. You should upload this as a separate file labeled 'Manuscript'.

If you would like to make changes to your financial disclosure, please include your updated statement in your cover letter. Guidelines for resubmitting your figure files are available below the reviewer comments at the end of this letter.

If applicable, we recommend that you deposit your laboratory protocols in protocols.io to enhance the reproducibility of your results. Protocols.io assigns your protocol its own identifier (DOI) so that it can be cited independently in the future. For instructions see: https://journals.plos.org/plosone/s/submission-guidelines#loc-laboratory-protocols. Additionally, PLOS ONE offers an option for publishing peer-reviewed Lab Protocol articles, which describe protocols hosted on protocols.io. Read more information on sharing protocols at https://plos.org/protocols?utm_medium=editorial-email&utm_source=authorletters&utm_campaign=protocols.

As the corresponding author, your ORCID iD is verified in the submission system and will appear in the published article. PLOS supports the use of ORCID, and we encourage all coauthors to register for an ORCID iD and use it as well. Please encourage your coauthors to verify their ORCID iD within the submission system before final acceptance, as unverified ORCID iDs will not appear in the published article. *Only*  the individual author can complete the verification step; PLOS staff *cannot*  verify ORCID iDs on behalf of authors.

We look forward to receiving your revised manuscript.

Kind regards,

Haiyang Chen

Academic Editor

PLOS One

Journal Requirements:

When submitting your revision, we need you to address these additional requirements.

1. Please ensure that your manuscript meets PLOS ONE's style requirements, including those for file naming. The PLOS ONE style templates can be found at

https://journals.plos.org/plosone/s/file?id=wjVg/PLOSOne_formatting_sample_main_body.pdf and

https://journals.plos.org/plosone/s/file?id=ba62/PLOSOne_formatting_sample_title_authors_affiliations.pdf.

2. Thank you for stating the following financial disclosure:

“This research was funded by Hanoi University of Science and Technology (HUST)

under project number T2024-PC-003. “

Please state what role the funders took in the study.  If the funders had no role, please state: "The funders had no role in study design, data collection and analysis, decision to publish, or preparation of the manuscript."

If this statement is not correct you must amend it as needed.

Please include this amended Role of Funder statement in your cover letter; we will change the online submission form on your behalf.

3. If the reviewer comments include a recommendation to cite specific previously published works, please review and evaluate these publications to determine whether they are relevant and should be cited. There is no requirement to cite these works unless the editor has indicated otherwise.

[Note: HTML markup is below. Please do not edit.]

Reviewers' comments:

Reviewer #2 Comments to the Author

Manuscript: PONE-D-26-13247

Summary

This study tests a conceptual model of AI-assisted academic cheating among 863 undergraduates in Vietnam. The topic is timely and the sample size is a clear strength. However, several issues with measurement validity, theoretical grounding, and result interpretation require substantial revision.

Major Comments

1. Discriminant validity (critical)

HTMT values among the four dependent constructs substantially exceed the 0.90 threshold: AIContentSub–NonDisclosureAI = 0.966, AITaskDelegation–AIPolicyViolation = 0.926, AITaskDelegation–NonDisclosureAI = 0.917, AIContentSub–AITaskDelegation = 0.905. These constructs are empirically indistinguishable in this sample. The authors must either consolidate the overlapping constructs or provide rigorous justification for retaining them as distinct. This is foundational — all downstream analyses are affected.

2. Theoretical grounding

All 16 hypotheses are drawn from a single qualitative study on postgraduate students, with no justification for applying them to undergraduates. The source model was explicitly exploratory and qualitative, not a validated quantitative framework. The authors should broaden the theoretical base or clearly justify the transferability of the original model to the present population and research design.

3. Overstated claims relative to effect sizes

The model explains only 3–6% of variance (R² = 0.034–0.055), and virtually all significant paths have effect sizes below the threshold for small (f² < 0.02). The manuscript — including the title — repeatedly describes institutional policy gaps as playing a 'prominent role.' This characterization is inconsistent with the data. All such language should be revised to reflect that findings are statistically significant but small in practical magnitude.

4. Gender–discipline confounding

Male students are concentrated in engineering while female students are concentrated in humanities and social sciences. Gender effects are reported without controlling for discipline, making it impossible to attribute the observed differences to gender rather than field of study. Discipline should be included as a covariate or the gender analysis should be conducted within disciplinary strata.

5. PLS-SEM justification

Given the large sample, confirmatory design, and explicit hypotheses, the choice of PLS-SEM over CB-SEM requires justification. The authors should explain why PLS-SEM is appropriate here, or consider whether CB-SEM is more defensible for a theory-testing study of this kind.

Minor Comments

Social desirability bias is acknowledged but its potential impact on path coefficient attenuation is not discussed. This should be elaborated in the discussion.

With 24 hypothesized paths, the risk of Type I error from multiple comparisons should be acknowledged and addressed.

Reviewer's Responses to Questions

**Comments to the Author**

1. Is the manuscript technically sound, and do the data support the conclusions?

Reviewer #1: Partly

**********

2. Has the statistical analysis been performed appropriately and rigorously?

Reviewer #1: Yes

**********

3. Have the authors made all data underlying the findings in their manuscript fully available?

The PLOS Data policy

Reviewer #1: Yes

**********

4. Is the manuscript presented in an intelligible fashion and written in standard English?

Reviewer #1: Yes

**********

Reviewer #1: This study focuses on the drivers of AI-assisted academic cheating among undergraduate students in the context of generative AI. The topic is practically relevant and has clear real-world value. The sample size is relatively large (N = 863), and the dataset has potential research value. However, the following issues remain:

1.The study’s target population is undergraduates, yet the four-category classification of cheating behaviors used in the paper is derived from qualitative findings from a postgraduate population (“AI-assisted academic cheating: a conceptual model based on postgraduate student voices,” DOI: 10.3389/fcomp.2025.1682190). The paper does not provide sufficient evidence that this four-dimensional structure shows clear empirical discriminant validity in an undergraduate sample. As indicated by the HTMT results in Table 6—AIContentSub–NonDisclosureAI = 0.966, AITaskDelegation–AIPolicyViolation = 0.926, AITaskDelegation–NonDisclosureAI = 0.917, and AIContentSub–AITaskDelegation = 0.905—HTMT values above 0.90 suggest these constructs overlap substantially in the undergraduate population and may effectively reflect the same concept, making it difficult to distinguish them clearly. It is recommended that highly redundant constructs be merged.

2.The study splits academic cheating into four distinct dimensions, but the theoretical framework does not offer differentiated mechanisms to explain why specific drivers would predict one type of cheating rather than another. All hypotheses are drawn from the findings of a single prior article, which results in 16 specific hypotheses that lack theoretical depth and amount to largely ad hoc variable testing. Moreover, as noted above, the cited article examines postgraduate students; applying its results directly to undergraduates without any justification is methodologically imprecise and difficult to defend.

3.In the limitations section, the authors acknowledge that the model has low explanatory power, yet the paper still uses wording such as “prominent role,” which does not align with the statistical results. The explained variance for cheating behaviors is R² = 0.034–0.055, accounting for only about 3%–6% of the variation. According to Cohen’s f² benchmarks, aside from PolicyGaps → AITaskDelegation (f² = 0.022), the effect sizes of the other significant paths are all below the threshold for a small effect, indicating extremely weak effects. It is recommended that the wording be revised to characterize the findings as “statistically significant but small in effect,” avoiding phrasing that could imply a strong influence.

4.The observed gender effects may be confounded by disciplinary/major-related variables. In the sample, 71.3% are male, and they are mainly from engineering/science fields, whereas most female participants are concentrated in humanities, education, social sciences, and related majors. The authors report a significant effect of gender on cheating behaviors but do not control for discipline/major in the structural model. Consider testing gender effects within disciplinary strata (i.e., stratifying by major/field) before reporting an association between gender and cheating; only if the effect remains significant should it be reported as related to cheating behavior.

5.The authors list social desirability bias as a limitation but do not further discuss its potential impact on the structural model estimates. Because academic cheating is a morally sensitive behavior, students may underreport or be reluctant to admit such behaviors, and many participants selected “never” for the relevant items. Restricted variance can attenuate estimated path coefficients, thereby reducing the model’s statistical power to detect potential relationships. The authors are encouraged to elaborate in the discussion on how this issue might affect path significance and effect-size estimation.

6.The current structural model is highly saturated, with four driving factors and two demographic variables simultaneously predicting four cheating dimensions, resulting in 24 hypothesized paths. This design resembles an all-inclusive enumeration model rather than a parsimonious theory-driven structural model. The authors should justify the current path specification more explicitly and consider simplifying the model after re-specifying the endogenous constructs.

7.The manuscript applies PLS-SEM to test a conceptual model, yet the rationale for choosing PLS-SEM is not sufficiently explained. Given the relatively large sample size (N = 863), the explicit hypotheses, and the confirmatory nature of the study, the authors should justify why PLS-SEM was preferred over covariance-based SEM approaches more commonly used for theory testing.

**********

what does this mean?). If published, this will include your full peer review and any attached files.

If you choose “no”, your identity will remain anonymous but your review may still be made public.

**Do you want your identity to be public for this peer review?**  For information about this choice, including consent withdrawal, please see our Privacy Policy

Reviewer #1: No

**********

[NOTE: If reviewer comments were submitted as an attachment file, they will be attached to this email and accessible via the submission site. Please log into your account, locate the manuscript record, and check for the action link "View Attachments". If this link does not appear, there are no attachment files.]

To ensure your figures meet our technical requirements, please review our figure guidelines: https://journals.plos.org/plosone/s/figures

You may also use PLOS’s free figure tool, NAAS, to help you prepare publication quality figures: https://journals.plos.org/plosone/s/figures#loc-tools-for-figure-preparation.

NAAS will assess whether your figures meet our technical requirements by comparing each figure against our figure specifications.

Attachment Submitted filename: PONE-D-26-13247_Review_Comments.docx

# Author comment: Author response to Decision Letter 1

**DOI:** 10.1371/journal.pone.0359549.r002

29 May 2026

RESPONSE TO EDITOR AND REVIEWERS

Dear Academic Editor and Reviewers,

We sincerely thank the Academic Editor, the reviewers, and the editorial team for their careful evaluation of our manuscript and for giving us the opportunity to revise and resubmit our work. We are grateful for the constructive comments, which helped us substantially improve the theoretical framing, methodological rigor, statistical analysis, and interpretation of the findings. We have carefully considered every comment and have revised the manuscript thoroughly. In particular, we changed the title, rewrote the abstract and introduction, replaced the original hypotheses with two research questions, restructured the Methods section, changed the analytical approach from PLS-SEM to EFA, CFA, and CB-SEM, and rewrote the Results and Discussion sections according to the revised empirical model. All major revisions in the manuscript have been highlighted in yellow for ease of review.

Response Academic Editor:

Thank you for submitting your manuscript 'Testing a Conceptual Model of Driving Factors of AI-Assisted Academic Cheating among Undergraduate Students' (PONE-D-26-13247) to PLOS ONE. We appreciate the effort invested in this work, and we recognize the relevance of the topic at a time when generative AI is reshaping academic integrity in higher education. After careful consideration of two independent reviewer reports, I am unable to accept the manuscript in its current form. However, given the strength of your dataset and the timeliness of the research question, I am willing to invite a major revision. I want to be transparent with you: this is a substantive revision with a high bar, and the outcome of the second round will depend critically on how the following concerns are addressed.

Response:

We sincerely thank the Academic Editor for the careful evaluation of our manuscript and for the opportunity to submit a major revision. We appreciate the recognition of the timeliness of the topic and the potential value of the dataset. We also fully understand that the revision required substantial methodological, theoretical, and interpretive changes. In response to the editor’s and reviewers’ concerns, we have made extensive revisions throughout the manuscript. The revised manuscript now has a new title, a rewritten abstract, a substantially revised introduction, two research questions instead of 24 hypotheses, a revised research design, a new participant distribution table, a clearer survey instrument section, a new data analysis strategy using EFA, CFA, and CB-SEM, and a fully rewritten Results, Discussion, Limitations, Practical Implications, and Future Research Directions section. Most importantly, we no longer present the study as a confirmatory test of a fixed conceptual model derived from prior qualitative research. Instead, the revised manuscript is positioned as an exploratory empirical study that first examines the latent structure of the item pool and then tests associations among the empirically identified constructs.

The single most important issue is discriminant validity. The HTMT values among your four cheating behavior constructs — several of which exceed 0.96 — indicate that these constructs are not empirically distinguishable in your undergraduate sample. Because the entire structural model is built around treating these as separate outcomes, this issue affects the validity of all downstream analyses. To move forward, the authors must either consolidate the overlapping constructs into a smaller, empirically defensible set, or provide compelling evidence — beyond the Fornell–Larcker criterion alone — that the four-category structure is justified. If this issue is not resolved to the reviewers' satisfaction, the manuscript will not be able to proceed further regardless of other improvements.

Response:

We agree that discriminant validity was the most critical issue in the original manuscript. The high HTMT values among the four AI-assisted cheating behavior constructs showed that these dimensions were not empirically distinguishable in the undergraduate sample. We therefore no longer retained the four-category cheating behavior structure as separate endogenous constructs. In the revised manuscript, we reanalyzed the full item pool using exploratory factor analysis without imposing the original conceptual categories. The EFA results showed that all eleven AI-assisted academic cheating behavior items loaded on a single factor. We therefore consolidated the four overlapping behavioral dimensions into one broader construct, labeled AI-Assisted Academic Cheating Behaviors (AICB). This factor structure was then evaluated using CFA within a CB-SEM framework. The revised measurement model also reports discriminant validity using both the Fornell–Larcker criterion and HTMT. Thus, the revised structural model is no longer built on empirically indistinguishable cheating behavior subdimensions.

Beyond this, the following concerns must also be addressed in the revision:

First, the theoretical framework derives all hypotheses from a single qualitative study on postgraduate students. The revision should either broaden the theoretical grounding or provide explicit justification for extending the model to an undergraduate population in a confirmatory quantitative design.

Response:

Thank you for this important comment. We agree that the previous version relied too heavily on one qualitative study conducted with postgraduate students and did not sufficiently justify the direct transfer of that model to an undergraduate sample. In response, we substantially revised the Introduction. In the revised manuscript, the prior qualitative study is used only as one source informing the development of the item pool, not as a validated conceptual model to be directly tested. We removed the previous claim that the study tested a conceptual model derived from prior qualitative research. We also removed all 24 hypotheses and replaced them with two broader research questions. This revision better reflects the exploratory nature of the study and avoids presenting the prior qualitative framework as a fully established quantitative model for undergraduate students. The revised Introduction now situates the study more broadly within the literature on academic integrity, generative AI, ethical ambiguity, technological affordances, and institutional guidance in higher education.

Second, the manuscript describes institutional policy gaps as playing a 'prominent role,' yet the explained variance is 3–6% and virtually all effect sizes fall below the conventional threshold for small. The language throughout — including the title — should be revised to accurately reflect the magnitude of the findings.

Response:

We agree that the previous wording overstated the strength of the findings. In particular, the phrase “prominent role” was not appropriate given the low explained variance and small effect sizes in the original model. We have therefore removed this phrasing from the title, abstract, Results, Discussion, and Implications.

The revised title is now:

Beyond Academic Pressure: Ethical Ambiguity, Technological Affordances, and Institutional Gaps as Enablers of AI-Assisted Academic Cheating among Undergraduate Students

This title reflects the revised empirical structure more accurately and avoids implying a strong causal or predictive effect. Throughout the revised manuscript, we now use more cautious wording such as “associated with,” “modest evidence of association,” and “small proportion of variance.” In the Results and Discussion sections, we explicitly state that the structural model explained only a small proportion of the variance in students’ self-reported AI-assisted academic cheating behaviors and that the findings should be interpreted as modest rather than strong explanatory evidence.

Third, the reported gender effects should be examined with discipline controlled, given the uneven distribution of male and female students across fields of study.

Response:

We appreciate this important concern. We agree that gender-related findings may be affected by the disciplinary composition of the sample, particularly because the study was conducted at a university of science and technology and the gender distribution was uneven. To address this issue more transparently, we revised the Participants section and added a new Table 1: Participant distribution by gender, student seniority, and field of study. This table shows the distribution of male and female students across STEM and non-STEM fields and clarifies the composition of the sample. Because the non-STEM subgroup was relatively small and the sample was not balanced across gender and field of study, we avoided making strong claims about gender as an independent explanatory factor. In the revised manuscript, gender is treated only as a relevant background variable, and the Discussion and Limitations sections now state clearly that the observed gender association was small, context-dependent, and should be interpreted cautiously. We also recommend that future studies examine gender effects using more balanced samples and disciplinary strata.

Finally, the rationale for choosing PLS-SEM over covariance-based SEM should be stated explicitly.

Response:

Thank you for raising this methodological concern. We agree that the original rationale for using PLS-SEM was insufficient, especially given the large sample size and the need to examine the measurement structure more rigorously. In response, we replaced the original PLS-SEM analysis with a new analytical strategy based on exploratory factor analysis, confirmatory factor analysis, and covariance-based structural equation modeling (CB-SEM). The revised Data Analysis section now explains this sequential procedure. First, EFA was used to identify the empirical structure of the full item pool without imposing the original conceptual categories. Second, CFA was used to evaluate the measurement model. Finally, CB-SEM was used to test the associations among the identified latent constructs and the roles of gender and student seniority. The Results section has been fully rewritten to report the EFA results, CFA model fit, reliability and validity evidence, discriminant validity, and the final structural model.

If you choose to revise, please submit a detailed point-by-point response letter that directly addresses each reviewer comment. Please note that a revision is not a guarantee of acceptance. The revised manuscript will be evaluated against the concerns above, and if the discriminant validity issue and the core analytical concerns remain unresolved, the manuscript will be declined at that stage.

We hope these comments are helpful and that you find a productive path forward. We look forward to hearing from you.

Response:

We are grateful for the editor’s clear guidance and for the opportunity to revise the manuscript. We believe that the revised manuscript addresses the central concerns raised in the decision letter. In particular, the discriminant validity problem has been resolved by consolidating the overlapping cheating behavior dimensions into a single empirically defensible construct; the theoretical framing has been repositioned from confirmatory model testing to exploratory empirical analysis; the language has been revised to avoid overstating effect sizes; the sample composition has been clarified; and the analytical strategy has been changed from PLS-SEM to EFA/CFA/CB-SEM. We hope that these revisions provide a stronger, more cautious, and more methodologically defensible manuscript.

Response to Journal Requirements:

When submitting your revision, we need you to address these additional requirements.

1. Please ensure that your manuscript meets PLOS ONE's style requirements, including those for file naming. The PLOS ONE style templates can be found at

https://journals.plos.org/plosone/s/file?id=wjVg/PLOSOne_formatting_sample_main_body.pdf and

https://journals.plos.org/plosone/s/file?id=ba62/PLOSOne_formatting_sample_title_authors_affiliations.pdf.

Response:

Thank you for this instruction. We have carefully revised the manuscript to comply with PLOS ONE’s style requirements, including formatting, manuscript structure, and file naming requirements.

2. Thank you for stating the following financial disclosure:

“This research was funded by Hanoi University of Science and Technology (HUST)

under project number T2024-PC-003. “

Please state what role the funders took in the study. If the funders had no role, please state: "The funders had no role in study design, data collection and analysis, decision to publish, or preparation of the manuscript."

If this statement is not correct you must amend it as needed.

Please include this amended Role of Funder statement in your cover letter; we will change the online submission form on your behalf.

Response:

Thank you for this clarification. We have revised the funding statement to include the role of the funder as required. The revised statement is: This research was funded by Hanoi University of Science and Technology (HUST) under project number T2024-PC-003. The funders had no role in study design, data collection and analysis, decision to publish, or preparation of the manuscript.

We have also included this amended Role of Funder statement in the cover letter.

3. If the reviewer comments include a recommendation to cite specific previously published works, please review and evaluate these publications to determine whether they are relevant and should be cited. There is no requirement to cite these works unless the editor has indicated otherwise.

Response:

Thank you for this note. The reviewers did not recommend any specific previously published works that must be cited. Therefore, no additional citations were added specifically in response to reviewer-recommended references. However, we revised the manuscript to ensure that the theoretical framing and discussion are appropriately supported by relevant literature.

If applicable, we recommend that you deposit your laboratory protocols in protocols.io to enhance the reproducibility of your results. Protocols.io assigns your protocol its own identifier (DOI) so that it can be cited independently in the future. For instructions see: https://journals.plos.org/plosone/s/submission-guidelines#loc-laboratory-protocols. Additionally, PLOS ONE offers an option for publishing peer-reviewed Lab Protocol articles, which describe protocols hosted on protocols.io. Read more information on sharing protocols at https://plos.org/protocols?utm_medium=editorial-email&utm_source=authorletters&utm_campaign=protocols.

Response: Not applicable. This study did not involve laboratory experiments or laboratory protocols. The study used a cross-sectional self-report survey, and the procedures for participant recruitment, survey administration, measurement, and statistical analysis are described in the Methods section. The dataset underlying the results is available through Figshare as stated in the Data Availability Statement.

Response to Reviewer #2

Manuscript: PONE-D-26-13247

Summary

This study tests a conceptual model of AI-assisted academic cheating among 863 undergraduates in Vietnam. The topic is timely and the sample size is a clear strength. However, several issues with measurement validity, theoretical grounding, and result interpretation require substantial revision.

Response:

We sincerely thank the reviewer for the careful and constructive evaluation of our manuscript. We agree that the previous version had important limitations related to discriminant validity, theoretical grounding, model specification, and interpretation of effect sizes. In response, we have made substantial revisions throughout the manuscript. The revised version includes a new title, a rewritten abstract, a revised introduction, two research questions instead of 24 hypotheses, a revised research design, a new participant distribution table, a clearer survey instrument section, a new data analysis strategy using EFA, CFA, and CB-SEM, and a fully rewritten Results, Discussion, and Implications section. These changes were made to better reflect the exploratory nature of the study and to avoid overstating the explanatory strength of the model.

Thank you for summarizing the strengths and concerns of the study. We appreciate the reviewer’s recognition of the timeliness of the topic and the value of the relatively large sample. We also agree that the original version required substantial revision, particularly in terms of measurement validity, theoretical framing, and interpretation. In response, we no longer present the study as a direct test of a conceptual model. Instead, the revised manuscript examines how perceived driving factors are associated with undergraduate students’ self-reported AI-assisted academic cheating behaviors. The revised analysis first explores the empirical factor structure of the item pool and then tests a more parsimonious structural model based on the identified constructs.

Major Comments

1. Discriminant validity (critical)

HTMT values among the four dependent constructs substantially exceed the 0.90 threshold: AIContentSub–NonDisclosureAI = 0.966, AITaskDelegation–AIPolicyViolation = 0.926, AITaskDelegation–NonDisclosureAI = 0.917, AIContentSub–AITaskDelegation = 0.905. These constructs are empirically indistinguishable in this sample. The authors must either consolidate the overlapping constructs or provide rigorous justification for retaining them as distinct. This is foundational — all downstream analyses are affected.

Response:

Thank you for identifying this critical issue. We agree that the four dependent constructs in the original model were not sufficiently distinguishable in the undergraduate sample. The high HTMT values among several behavioral dimensions indicated that these constructs overlapped substantially and should not be retained as separate endogenous variables. To address this issue, we fully reanalyzed the measurement structure. In the revised manuscript, we used exploratory factor analysis to examine the full item pool without imposing the original four-category structure. The EFA results showed that all eleven AI-assisted cheating behavior items loaded on a single factor. Therefore, we consolidated the four overlapping behavioral dimensions into one broader construct, labeled AI-Assisted Academic Cheating Behaviors (AICB). This structure was then validated through CFA within a CB-SEM framework. The revised model therefore no longer depends on empirically indistinguishable cheating subdimensions.

2. Theoretical grounding

All 16 hypotheses are drawn from a single qualitative study on postgraduate students, with no justification for applying them to undergraduates. The source model was explicitly exploratory and qualitative, not a validated quantitative framework. The authors should broaden the theoretical base or clearly justify the transferability of the original model to the present population and research design.

Response:

We agree with the reviewer’s concern. In the previous version, the hypotheses were too heavily derived from a single qualitative study on postgraduate students, and the manuscript did not sufficiently justify the transfer of that exploratory model to an undergraduate sample. We have therefore revised the Introduction substantially. In the revised version, we no longer claim to test a conceptual model derived from the prior qualitative study. We also removed all 24 hypotheses and replaced them with two broader research questions. This revision more accurately reflects the exploratory and empirical purpose of the study. The prior qualitative study is now used only as one source informing the development of the item pool, not as a validated theoretical framework to be directly tested. We also broadened the framing by situating the study within wider literature on academic integrity, AI-assisted cheating, ethical ambiguity, technological affordances, and institutional guidance in higher education.

3. Overstated claims relative to effect sizes

The model explains only 3–6% of variance (R² = 0.034–0.055), and virtually all significant paths have effect sizes below the threshold for small (f² < 0.02). The manuscript — including the title — repeatedly describes institutional policy gaps as playing a 'prominent role.' This characterization is inconsistent with the data. All such language should be revised to reflect that findings are statistically significant but small in practical magnitude.

Response:

Thank you for this important comment. We agree that the previous wording, especially the phrase “prominent role,” overstated the practical magnitude of the findings. In response, we revised the title, abstract, Results, Discussion, and Implications to avoid language that implies strong prediction or strong explanatory power. The revised manuscript now uses more cautious wording, such as “associated with,” “modest evidence of association,” and “small proportion of variance.” We also explicitly state that the structural model explained only a small proportion of the variance in self-reported AI-assisted academic cheating behaviors and that the findings should be interpreted as modest rather than strong explanatory evidence. The new title no longer claims a “prominent role” of institutional policy gaps; instead, it highlights the broader pattern of ethical ambiguity, technological affordances, and institutional gaps as perceived enabling conditions.

4. Gender–discipline confounding

Male students are concentrated in engineering while female students are concentrated in humanities and social sciences. Gender effects are reported without controlling for discipline, making it impossible to attribute the observed differences to gender rather than field of study. Discipline should be included as a covariate or the gender analysis should be conducted within disciplinary strata.

Response:

We appreciate this valuable point. We agree that gender-related findings may be affected by the disciplinary composition of the sample, particularly because the study was conducted at a university of science and technology and the gender distribution was uneven. To make the sample composition more transparent, we revised the Participants section and added a new table showing participant distribution by gender, student seniority, and field of study. This table clarifies that most participants were from STEM fields, and that both male and female students were represented in STEM and non-STEM groups, although the distribution remained uneven. Because the non-STEM subgroup was relatively small and the sample was not balanced across gender and discipline, we did not conduct strong discipline-stratified claims in the revised manuscript. Instead, gender is now treated as a relevant background variable, and the Discussion and Limitations sections explicitly state that the gender association was small, context-dependent, and should be interpreted cautiously. We also note that future research should examine gender effects in more balanced samples and across disciplinary strata.

5. PLS-SEM justification

Given the large sample, confirmatory design, and explicit hypotheses, the choice of PLS-SEM over CB-SEM requires justification. The authors should explain why PLS-SEM is appropriate here, or consider whether CB-SEM is more defensible for a theory-testing study of this kind.

Response:

Thank you for raising this methodological concern. We agree that the use of PLS-SEM in the original version was not sufficiently justified, particularly given the large sample size and the need to evaluate the measurement structure more rigorously. In response, we replaced the PLS-SEM analysis with a new analytical strategy based on exploratory factor analysis, confirmatory factor analysis, and covariance-based structural equation modeling (CB-SEM). The revised Data Analysis section now explains this sequential approach. EFA was first used to explore the empirical structure of the full item pool. CFA was then used to validate the measurement model. Finally, CB-SEM was used to test the associations among the identified latent constructs and the roles of gender and student seniority. The Results section has been fully rewritten to report EFA, CFA, discriminant validity, model fit, and structural path results under this revised CB-SEM framework.

Minor Comments

Social desirability bias is acknowledged but its potential impact on path coefficient attenuation is not discussed. This should be elaborated in the discussion.

Response:

Thank you for this helpful suggestion. We agree that social desirability bias may affect not only the reported level of AI-assisted academic cheating behaviors but also the estimated path coefficients in the structural model. Because academic cheating is a morally sensitive behavior, students may underreport their actual engagement, even in an anonymous survey. This may restrict the variance of the behavioral construct, attenuate path coefficients, reduce statistical power, and make some potential associations more difficult to detect. We have expanded the Limitations section to discuss this issue more explicitly. The revised manuscript now states that the low mean scores and frequent “never” responses may have restricted the variance of the dependent construct and may partly explain the small explained variance and non-significant paths. We also recommend future studies using methods that can reduce self-report bias, such as list experiments, indirect questioning, mixed-methods designs, or behavioral data where feasible.

With 24 hypothesized paths, the risk of Type I error from multiple comparisons should be acknowledged and addressed.

Response:

We agree with the reviewer that the previous model, with 24 hypothesized paths, increased the risk of Type I error and contributed to an overly complex model specification. This issue has been addressed through a major revision of the analytical model. In the revised manuscript, we removed the 24 hypotheses and replaced them with two broader research questions. The structural model was also simplified substantially. Instead of four driving factors and two demographic variables predicting four separate cheating dimensions, the revised model includes one endogenous construct, AI-Assisted Academic Cheating Behaviors (AICB), and four direct paths from ETIG, APPC, gender, and student seniority. This more parsimonious specification reduces the multiple-comparison problem and better reflects the exploratory nature of the study.

Response to Reviewer #1:

This study focuses on the drivers of AI-assisted academic cheating among undergraduate students in the context of generative AI. The topic is practically relevant and has clear real-world value. The sample size is relatively large (N = 863), and the dataset has potential research value. However, the following issues remain:

Response:

We sincerely thank the reviewer for the constructive and detailed comments. We agree that the previous version had several important methodological and interpretive limitations, particularly regarding the use of a postgraduate-derived conceptual model, the discriminant validity of the four cheating behavior dimensions, the large number of hypothesized paths, the use of PLS-SEM, and the interpretation of small effects. In response, we have substantially revised the manuscript. The revised version now has a new title, a rewritten abstract, a revised introduction, two research questions instead of 24 hypotheses, a redesigned Methods section, a new participant distribution table, a clearer description of the survey instrument, a new data analysis strategy based on EFA, CFA, and CB-SEM, a fully rewritten Results section, and a revised Discussion and Implications section. These changes were made to better reflect the exploratory nature of the study and to avoid overstating the explanatory power of the model.

1.The study’s target population is undergraduates, yet the four-category classification of cheating behaviors used in the paper is derived from qualitative findings from a postgraduate population (“AI-assisted academic cheating: a conceptual model based on postgraduate student voices,” DOI: 10.3389/fcomp.2025.1682190). The paper does not provide sufficient evidence that this four-dimensional structure shows clear empirical discriminant validity in an undergraduate sample. As indicated by the HTMT results in Table 6—AIContentSub–NonDisclosureAI = 0.966, AITaskDelegation–AIPolicyViolation = 0.926, AITaskDelegation–NonDisclosureAI = 0.917, and AIContentSub–AITaskDelegation = 0.905—HTMT values above 0.90 suggest these constructs overlap substantially in the undergraduate population and may effectively reflect the same concept, making it difficult to distinguish them clearly. It is recommended that highly redundant constructs be merged.

Response:

Thank you for this important observation. We agree that the original four-category classification of AI-assisted academic cheating behaviors did not show sufficient empirical discriminant validity in the undergraduate sample. As the reviewer noted, several HTMT values among the four behavioral dimensions were above .90, indicating substantial overlap. In response, we no longer treated these four categories as separate endogenous constructs. In the revised manuscript, we reanalyzed the full item pool using exploratory factor analysis. The results showed that all eleven AI-assisted cheating behavior items loaded on a single factor, which we labeled AI-Assisted Academic Cheating Behaviors (AICB). We then validated this structure using CFA within a CB-SEM framework. This revision directly addresses the reviewer’s concern by merging the highly overlapping behavioral dimensions into one broader behavioral construct rather than forcing an unsupported four-dimensional structure.

2.The study splits academic cheating into four distinct dimensions, but the theoretical framework does not offer differentiated mechanisms to explain why specific drivers would predict one type of cheating rather than another. All hypotheses are drawn from the findings of a single prior article, which results in 16 specific hypotheses that lack theoretical depth and amount to largely ad hoc variable testing. Moreover, as noted above, the cited article examines postgraduate students; applying its results directly to undergraduates without any justification is methodologically imprecise and difficult to defend.

Response:

We agree with the reviewer’s concern. In the previous version, the hypotheses were too extensive and were mainly derived from one prior qualitative study. This created the impression of ad hoc variable testing and did not provide a sufficiently differentiated theoretical mechanism for why each driver should predict each specific type of cheating behavior. To address this issue, we substantially revised the Introduction. We removed the framing that the study was directly testing a conceptual model derived from the prior qualitative article. We also removed all hypotheses and replaced them with two broader research questions. This change reflects the actual nature of the study more accurately: it is exploratory and empirical, first examining the factor structure of the item pool and then testing associations among the identified constructs. The revised study no longer claims to test a fixed conceptual model, but instead examines how perceived driving factors are associated with students’ self-reported AI-assisted academic cheating behaviors.

3.In the limitations section, the authors acknowledge that the model has low explanatory power, yet the paper still uses wording such as “prominent role,” which does not align with the statistical results. The explained variance for cheating behaviors is R² = 0.034–0.055, accounting for only about 3%–6% of the variation. According to Cohen’s f² benchmarks, aside from PolicyGaps → AITaskDelegation (f² = 0.022), the effect sizes of the other significant paths are all below the threshold for a small effect, indicating extremely weak effects. It is recommended that the wording be revised to characterize the findings as “statistically significant but small in effect,” avoiding phrasing that could imply a strong influence.

Response:

Thank you for pointing this out. We agree that the wording in the previous version overstated the strength of the findings. In the revised manuscript, we removed wording such as “prominent role” and avoided language that implies strong influence or strong prediction. The title, abstract, Results, Discussion, and Implications have all been revised to use more cautious wording, such as “associated with,” “modest evidence of association,” and “small proportion of the variance.” In addition, because the model was re-specified, the previous interpretation of multiple small PLS-SEM path effects was no longer retained. The revised CB-SEM model reports one endogenous construct, AICB, and clearly states that the model explained only a small proportion of the variance. We also explicitly note that the findings should be interpreted as modest rather than strong explanatory evidence.

4.The observed gender effects may be confounded by disciplinary/major-related variables. In the sample, 71.3% are male, and they are mainly from engineering/science fields, whereas most female participants are concentrated in humanities, education, social sciences, and related majors. The authors report a significant effect of gender on cheating behaviors but do not control for discipline/major in the structural model. Consider testing gender effects within disciplinary strata (i.e., stratifying by major/field) before reporting an association between gender and cheating; only if the effect remains significant should it be reported as related to cheating behavior.

Response:

We appreciate this important comment. We agree that gender-related findings should be interpreted carefully because the sample was drawn from a university of science and technology and was unevenly distributed by gender. To make the sample structure more transparent, we revised the Participants section and added a new Table 1: Participant distribution by gender, student seniority, and field of study. This table reports the cross-tabulation of gender and student seniority by STEM and non-STEM fields. The revised table shows that both male and female students were concentrated mainly in STEM fields, although the overall gender distribution remained uneven. Because the non-STEM subgroup was relatively small and the sample was not balanced across field and gender, we did not overinterpret gender as an independent explanatory factor. Instead, gender is now treated as a relevant background variable. We also revised the Discussion and Limitations sections to state clearly that the gender association was small, context-dependent, and should be interpreted cautiously. We further acknowledge that future studies should test gender effects in more balanced samples and across disciplinary strata.

5.The authors list social desirability bias as a limitation but do not further discuss its potential impact on the structural model estimates. Because academic cheating is a morally sensitive behavior, students may underreport or be reluctant to admit such behaviors, and many participants selected “never” for the relevant items. Restricted variance can attenuate estimated path coefficients, thereby reducing the model’s statistical power to detect potential relationships. The authors are encouraged to elaborate in the discussion on how this issue might affect path significance and effect-size estimation.

Response:

Thank you for this helpful suggestion. We agree that social desirability bias may affect not only the reported level of AI-assisted academic cheating behaviors but also the estimated relationships in the structural model. Because academic cheating is a morally sensitive topic, students may underreport their actual behaviors even in an anonymous survey. This can restrict the variance of the dependent construct and attenuate path coefficients, thereby reducing the model’s power to detect potential associations. In response, we expanded the Limitations section. We now explain that the relatively low level of self-reported cheating behaviors and the frequent “never” responses may have restricted variance in the behavioral construct. We also note that this may have weakened the estimated path coefficients and contributed to the small explained variance and non-significant paths. The revised manuscript therefore interprets both significant and non-significant paths more cautiously and recommends future studies using methods that reduce self-report bias, such as list experiments, indirect questioning, mixed-methods designs, or behavioral data where feasible.

6.The current structural model is highly saturated, with four driving factors and two demographic variables simultaneously predicting four cheating dimensions, resulting in 24 hypothesized paths. This design resembles an all-inclusive enumeration model rather than a parsimonious theory-driven structural model. The authors should justify the current path specification more explicitly and consider simplifying the model after re-specifying the endogenous constructs.

Response:

We agree with the reviewer’s assessment. The previous structural model included four driving factors and two demographic variables predicting four behavioral dimensions, resulting in 24 hypothesized paths. This made the model overly complex and insufficiently parsimonious. In the revised manuscript, we simplified the model substantially. After reanalyzing the item pool using EFA and CFA, the four overlapping behavioral dimensions were merged into one broader construct: AI-Assisted Academic Cheating Behaviors (AICB). The perceived driving factors were also reorganized into two broader constructs: Ethical Ambiguity, Technological Affordances, and Institutional Gaps (ETIG) and Academic Pressure and Peer Comparison (APPC). The revised structural model therefore includes only four direct paths to AICB: ETIG, APPC, gender, and student seniority. This new model is more parsimonious, better aligned with the empirical factor structure, and more appropriate for the exploratory nature of the study.

7.The manuscript applies PLS-SEM to test a conceptual model, yet the rationale for choosing PLS-SEM is not sufficiently explained. Given the relatively large sample size (N = 863), the explicit hypotheses, and the confirmatory nature of the study, the authors should justify why PLS-SEM was preferred over covariance-based SEM approaches more commonly used for theory testing.

Response:

Thank you for raising this methodological issue. We agree that the original use of PLS-SEM was not sufficiently justified, especially given the large sample size and the need to evaluate the measurement structure more rigorously. In response, we replaced the PLS-SEM analysis with a new analytical strategy based on exploratory factor analysis, confirmatory factor analysis, and covariance-based structural equation modeling (CB-SEM). The revised Data Analysis section now explains this sequential procedure. EFA was first used to identify the empirical factor structure of the full item pool without imposing the original conceptual categories. CFA was then used to validate the measurement model. Finally, CB-SEM was used to test the associations among the identified latent constructs and the roles of gender and student seniority. The Results section has been fully rewritten according to this revised analytical strategy, including EFA results, CFA model fit, reliability and validity evidence, discriminant validity using both Fornell–Larcker and HTMT, and the final structural model results.

Once again, we sincerely thank the Academic Editor and reviewers for their time, expertise, and constructive guidance. We believe that the revised manuscript is substantially improved in terms of theoretical clarity, methodological appropriateness, analytical rigor, and cautious interpretation of the findings. We hope that the revised version and the detailed point-by-point responses adequately address all concerns raised during the review process. We respectfully submit the revised manuscript for your further consideration.

Sincerely,

Hanh Van Nguyen

Faculty of Education, Hanoi University of Science and Technology, Hanoi, Vietnam

Email: hanh.nguyenvan@hust.edu.vn

Attachment Submitted filename: Respond to Reviewers.pdf

# Peer review: Decision Letter 1

**DOI:** 10.1371/journal.pone.0359549.r003

**Authors:** Haiyang Chen

23 Aug 2026

Thank you for submitting your manuscript to PLOS One. After careful consideration, we feel that it has merit but does not fully meet PLOS One’s publication criteria as it currently stands. Therefore, we invite you to submit a revised version of the manuscript that addresses the points raised during the review process.

Required Revisions

Please address the following items in your next revision:

1. Control for field of study. Reporting the gender distribution across STEM and non-STEM fields does not rule out confounding. You must incorporate appropriate statistical control and report how the gender–AICB association changes after adjustment.
2. Temper causal language. Despite acknowledging the cross-sectional design, the manuscript still contains phrasing that implies causality (e.g., in the title, discussion, and implications). Revise the language throughout to reflect association rather than causation.
3. Align Table 11 terminology. Your revised framework uses research questions, yet Table 11 retains hypothesis-testing terminology such as "hypothesis testing" and "Supported/Not supported." Revise this table to be consistent with your exploratory research question framework.

Your revisions thus far have partially addressed these points, but the remaining issues listed above must be conclusively resolved. I require a clear, point-by-point explanation of how the current revision satisfies each of these original requirements and the reviewer's new concerns, so that no outstanding issues remain

Please submit your revised manuscript by Oct 07 2026 11:59PM. If you will need more time than this to complete your revisions, please reply to this message or contact the journal office at plosone@plos.org. When you're ready to submit your revision, log on to https://www.editorialmanager.com/pone/ and select the 'Submissions Needing Revision' folder to locate your manuscript file.

- A letter that responds to each point raised by the academic editor and reviewer(s). You should upload this letter as a separate file labeled 'Response to Reviewers'.
- A marked-up copy of your manuscript that highlights changes made to the original version. You should upload this as a separate file labeled 'Revised Manuscript with Track Changes'.
- An unmarked version of your revised paper without tracked changes. You should upload this as a separate file labeled 'Manuscript'.

If you would like to make changes to your financial disclosure, please include your updated statement in your cover letter. Guidelines for resubmitting your figure files are available below the reviewer comments at the end of this letter.

If applicable, we recommend that you deposit your laboratory protocols in protocols.io to enhance the reproducibility of your results. Protocols.io assigns your protocol its own identifier (DOI) so that it can be cited independently in the future. For instructions see: https://journals.plos.org/plosone/s/submission-guidelines#loc-laboratory-protocols. Additionally, PLOS One offers an option for publishing peer-reviewed Lab Protocol articles, which describe protocols hosted on protocols.io. Read more information on sharing protocols at https://plos.org/protocols?utm_medium=editorial-email&utm_source=authorletters&utm_campaign=protocols.

As the corresponding author, your ORCID iD is verified in the submission system and will appear in the published article. PLOS supports the use of ORCID, and we encourage all coauthors to register for an ORCID iD and use it as well. Please encourage your coauthors to verify their ORCID iD within the submission system before final acceptance, as unverified ORCID iDs will not appear in the published article. *Only*  the individual author can complete the verification step; PLOS staff *cannot*  verify ORCID iDs on behalf of authors.

We look forward to receiving your revised manuscript.

Kind regards,

Haiyang Chen

Academic Editor

PLOS One

Journal Requirements:

If the reviewer comments include a recommendation to cite specific previously published works, please review and evaluate these publications to determine whether they are relevant and should be cited. There is no requirement to cite these works unless the editor has indicated otherwise.

Please review your reference list to ensure that it is complete and correct. If you have cited papers that have been retracted, please include the rationale for doing so in the manuscript text, or remove these references and replace them with relevant current references. Any changes to the reference list should be mentioned in the rebuttal letter that accompanies your revised manuscript. If you need to cite a retracted article, indicate the article’s retracted status in the References list and also include a citation and full reference for the retraction notice.

Additional Editor Comments:

[Note: HTML markup is below. Please do not edit.]

Reviewers' comments:

Reviewer's Responses to Questions

**Comments to the Author**

Reviewer #1: All comments have been addressed

**********

2. Is the manuscript technically sound, and do the data support the conclusions?

Reviewer #1: Yes

**********

3. Has the statistical analysis been performed appropriately and rigorously?

Reviewer #1: Yes

**********

4. Have the authors made all data underlying the findings in their manuscript fully available?

The PLOS Data policy

Reviewer #1: Yes

**********

5. Is the manuscript presented in an intelligible fashion and written in standard English?

Reviewer #1: Yes

**********

Reviewer #1: Dear Authors,

This study examines perceived factors associated with AI-assisted academic cheating among undergraduate students. The topic is timely and relevant, and the large sample is a clear strength. Compared with the original submission, the revised manuscript has improved considerably, particularly in the clarity with which the factor structure, sample characteristics, and study limitations are reported. However, several aspects of the interpretation and reporting of the findings require further clarification and revision.

1. The revised manuscript now reports the gender distribution across STEM and non-STEM fields, which improves the transparency of the sample. However, this descriptive information does not rule out potential confounding by field of study. The authors should adjust for field of study in the structural model or provide a sensitivity analysis stratified by STEM versus non-STEM status, and report whether the association between gender and AICB changes meaningfully after adjustment. If the association is substantially attenuated or becomes unstable, the interpretation of the gender finding should be tempered accordingly.

2. The revised manuscript appropriately acknowledges that the cross-sectional design does not support causal inference. However, relatively strong causal language remains in the title, discussion, and practical implications. Given the cross-sectional, self-report design, the authors should further temper this wording to avoid implying causality.

3. The terminology in Table 11 is inconsistent with the revised research framework. The revised manuscript is now structured around research questions, whereas Table 11 still uses hypothesis-testing terms such as “hypothesis testing” and “Supported/Not supported.” Please revise the terminology in Table 11 to ensure consistency with the research questions and the exploratory framing of the revised manuscript.

**********

what does this mean?). If published, this will include your full peer review and any attached files.

If you choose “no”, your identity will remain anonymous but your review may still be made public.

**Do you want your identity to be public for this peer review?**  For information about this choice, including consent withdrawal, please see our Privacy Policy

Reviewer #1: No

**********

[NOTE: If reviewer comments were submitted as an attachment file, they will be attached to this email and accessible via the submission site. Please log into your account, locate the manuscript record, and check for the action link "View Attachments". If this link does not appear, there are no attachment files.]

To ensure your figures meet our technical requirements, please review our figure guidelines: https://journals.plos.org/plosone/s/figures

You may also use PLOS’s free figure tool, NAAS, to help you prepare publication quality figures: https://journals.plos.org/plosone/s/figures#loc-tools-for-figure-preparation.

NAAS will assess whether your figures meet our technical requirements by comparing each figure against our figure specifications.

# Author comment: Author response to Decision Letter 2

**DOI:** 10.1371/journal.pone.0359549.r004

29 Aug 2026

RESPONSE TO EDITOR AND REVIEWERS

Dear Academic Editor and Reviewers,

We sincerely thank the Academic Editor, Reviewer 1, and the editorial team for their careful re-evaluation of our manuscript and for the opportunity to revise it again. We have addressed the remaining concerns point by point. In this revision, we incorporated field of study into the structural model as an observed control variable and re-estimated the CB-SEM model; systematically revised the title, abstract, introduction, discussion, and implications to remove causal framing; and fully aligned Table 11 with the research-question framework. We also updated the affected Methods and Results sections, figures, tables, limitations, and references. All revisions are shown in the marked manuscript using Track Changes.

Response Academic Editor:

Thank you for submitting your manuscript to PLOS One. After careful consideration, we feel that it has merit but does not fully meet PLOS One’s publication criteria as it currently stands. Therefore, we invite you to submit a revised version of the manuscript that addresses the points raised during the review process.

Response:

Thank you for the opportunity to submit a further revision. We have carefully addressed each of the remaining required revisions and Reviewer 1's concerns. The principal changes are: inclusion of field of study as an observed control variable in the structural model; re-estimation and full reporting of the revised CB-SEM results; systematic removal of causal wording throughout the manuscript; and revision of Table 11 so that it reports structural associations rather than hypothesis testing. The revised manuscript also updates all dependent sections so that the Methods, Results, Discussion, practical implications, and limitations are internally consistent. Detailed responses to each point are provided below.

Required Revisions

Please address the following items in your next revision:

Control for field of study. Reporting the gender distribution across STEM and non-STEM fields does not rule out confounding. You must incorporate appropriate statistical control and report how the gender–AICB association changes after adjustment.

Response:

We agree that descriptive reporting of STEM/non-STEM composition is not sufficient to address potential confounding. Field of study is now included directly in the structural model as an observed control variable, coded 0 = STEM and 1 = non-STEM. Gender was coded 0 = female and 1 = male, and student seniority was retained as an observed background variable. The coding and role of field of study are now described in Sections 2.2-2.4, and the revised model is reported in Section 3.4, Fig. 3, Table 10, and Table 11. The revised structural model showed acceptable fit, chi-square (237) = 1013.849, chi-square/df = 4.278, CFI = .924, TLI = .912, RMSEA = .062 (90% CI [.058, .066]), and SRMR = .0523. Field of study was positively associated with AICB (beta = .104, p = .008); given the coding, non-STEM students tended to report higher AICB scores than STEM students. The Gender -> AICB coefficient was beta = .183 (p = .002) in the model reported before field of study was introduced and beta = .142 (p < .001) in the current model including field of study. Thus, the gender association was not attenuated after field of study was included; it remained statistically significant and modest in magnitude. We have nevertheless tempered the interpretation because the sample remains unevenly distributed across gender and disciplinary groups. The revised interpretation is reflected in the Abstract, Results, Discussion (especially RQ2), practical implications, and limitations.

Temper causal language. Despite acknowledging the cross-sectional design, the manuscript still contains phrasing that implies causality (e.g., in the title, discussion, and implications). Revise the language throughout to reflect association rather than causation.

Response:

We agree and have systematically revised the manuscript to ensure that the study is framed in terms of association rather than causation. Most visibly, the title has been changed from the earlier causal formulation using 'Enablers' to: 'Perceived factors associated with self-reported AI-assisted academic cheating among undergraduate students: Evidence from ethical ambiguity, technological affordances, institutional gaps, academic pressure, and peer comparison' (p. 1). Across the Abstract, Introduction, Methods, Results, Discussion, and practical implications, study-specific terms such as 'driving factors,' 'enablers,' 'direct effects,' and other causal formulations were replaced with language such as 'associated with,' 'relationship,' and 'factors associated with.' The Discussion now explicitly states that the findings are evidence of association rather than strong explanatory or causal evidence. We also added a specific caution regarding the negative APPC coefficient: it is not interpreted as evidence that academic pressure or peer comparison reduces cheating. The practical implications are now framed as areas institutions may consider, rather than as interventions demonstrated to reduce AICB. Finally, the limitations explicitly state that the cross-sectional design does not establish temporal ordering or causal relationships.

Align Table 11 terminology. Your revised framework uses research questions, yet Table 11 retains hypothesis-testing terminology such as "hypothesis testing" and "Supported/Not supported." Revise this table to be consistent with your exploratory research question framework.

Response:

Thank you. Table 11 has been fully revised to align with the research-question framework. Its title is now 'Structural path estimates for associations with self-reported AI-assisted academic cheating behaviors'. The former 'Result' column and the labels 'Supported/Not supported' have been removed, as has the note defining when a hypothesis is supported. The table now reports only the path, standardized coefficient (beta), standard error (SE), critical ratio (CR), and p value. The accompanying text interprets these estimates as statistical associations. This terminology is now consistent with RQ1 and RQ2 and with the exploratory framing used throughout the manuscript.

Your revisions thus far have partially addressed these points, but the remaining issues listed above must be conclusively resolved. I require a clear, point-by-point explanation of how the current revision satisfies each of these original requirements and the reviewer's new concerns, so that no outstanding issues remain

Response:

We appreciate this instruction and have structured the present rebuttal accordingly. Each remaining issue is addressed separately with (i) the specific revision made, (ii) the relevant quantitative result where applicable, and (iii) the location of the change in the revised manuscript. In particular, the field-of-study concern is resolved through direct statistical control and explicit reporting of the Gender -> AICB coefficient before and after inclusion of field of study; causal language has been revised throughout the manuscript rather than only in isolated sections; and Table 11 has been fully converted from hypothesis-testing terminology to association-based reporting. The corresponding responses to Reviewer 1 below provide the same point-by-point clarification so that the editor and reviewer can readily verify each revision.

Response to Journal Requirements:

If the reviewer comments include a recommendation to cite specific previously published works, please review and evaluate these publications to determine whether they are relevant and should be cited. There is no requirement to cite these works unless the editor has indicated otherwise.

Response:

No reviewer in this round requested that specific publications be cited. We therefore did not add citations solely in response to a citation request. We re-evaluated the literature used to support the revised framing and retained citations only where they were relevant to the claims made in the manuscript.

Please review your reference list to ensure that it is complete and correct. If you have cited papers that have been retracted, please include the rationale for doing so in the manuscript text, or remove these references and replace them with relevant current references. Any changes to the reference list should be mentioned in the rebuttal letter that accompanies your revised manuscript. If you need to cite a retracted article, indicate the article’s retracted status in the References list and also include a citation and full reference for the retraction notice.

Response:

We reviewed the reference list for completeness, bibliographic accuracy, and retraction status. Several entries were corrected or updated during revision, including the DOI and publication details for the Fornell-Larcker reference, the DOI for the Narra Journal article, updated final publication information for the Journal of Applied Research in Higher Education article, and the DOI for the DASS-21 reference. We also checked the cited literature for retraction notices and did not identify a retracted publication among the references retained in the manuscript. No retracted article is cited as evidence in the revised manuscript.

Response to Reviewer 1

This study examines perceived factors associated with AI-assisted academic cheating among undergraduate students. The topic is timely and relevant, and the large sample is a clear strength. Compared with the original submission, the revised manuscript has improved considerably, particularly in the clarity with which the factor structure, sample characteristics, and study limitations are reported. However, several aspects of the interpretation and reporting of the findings require further clarification and revision.

Response:

Thank you for recognizing the improvements in the revised manuscript and for identifying the remaining issues requiring clarification. We have addressed each of the three concerns in the current revision. In particular, we have controlled for field of study in the structural model, further removed causal wording across the manuscript, and revised Table 11 to align completely with the research-question framework. Our detailed responses are provided below.

1. The revised manuscript now reports the gender distribution across STEM and non-STEM fields, which improves the transparency of the sample. However, this descriptive information does not rule out potential confounding by field of study. The authors should adjust for field of study in the structural model or provide a sensitivity analysis stratified by STEM versus non-STEM status, and report whether the association between gender and AICB changes meaningfully after adjustment. If the association is substantially attenuated or becomes unstable, the interpretation of the gender finding should be tempered accordingly.

Response:

We agree. Field of study is now included as an observed control variable in the structural model (0 = STEM, 1 = non-STEM), rather than being treated only descriptively. The revised model is presented in Fig. 3 and Tables 10-11, and the relevant coding and analytical treatment are described in Sections 2.2-2.4. The model including field of study showed acceptable fit: chi-square(237) = 1013.849, chi-square/df = 4.278, CFI = .924, TLI = .912, RMSEA = .062 (90% CI [.058, .066]), and SRMR = .0523. Field of study was associated with AICB (beta = .104, p = .008), with non-STEM students tending to report higher AICB scores than STEM students. Importantly, the Gender -> AICB association was beta = .183 (p = .002) in the model reported before field of study was introduced and beta = .142 (p < .001) in the current model including field of study. Therefore, the gender association remained statistically significant and was not attenuated. We have nevertheless interpreted this association cautiously because the sample was unevenly distributed by gender and field of study and came from a single institution. This caution is now explicit in the Discussion and Limitations.

2. The revised manuscript appropriately acknowledges that the cross-sectional design does not support causal inference. However, relatively strong causal language remains in the title, discussion, and practical implications. Given the cross-sectional, self-report design, the authors should further temper this wording to avoid implying causality.

Response:

We agree and have further tempered the language throughout the manuscript. The title no longer uses 'Enablers' and now explicitly uses 'factors associated with.' Study-specific causal terms such as 'driving factors,' 'direct effects,' and causal descriptions of the findings were replaced with association-based wording. The Abstract and Results report statistical associations; the Discussion explicitly states that the findings should not be interpreted as causal evidence; and the practical implications are framed as areas for institutional attention rather than demonstrated intervention effects. We also explicitly state that the negative APPC coefficient should not be interpreted as evidence that academic pressure protects against or reduces AI-assisted cheating. The cross-sectional limitation is reiterated in Section 4.3.

3. The terminology in Table 11 is inconsistent with the revised research framework. The revised manuscript is now structured around research questions, whereas Table 11 still uses hypothesis-testing terms such as “hypothesis testing” and “Supported/Not supported.” Please revise the terminology in Table 11 to ensure consistency with the research questions and the exploratory framing of the revised manuscript.

Response:

Thank you. Table 11 has been revised to remove all hypothesis-testing terminology. It is now titled 'Structural path estimates for associations with self-reported AI-assisted academic cheating behaviors.' The 'Supported/Not supported' judgments and the hypothesis-support note have been removed. The table now reports Path, beta, SE, CR, and p only, and the surrounding text discusses the coefficients as associations relevant to RQ1 and RQ2. This revision makes Table 11 fully consistent with the exploratory research-question framework.

Thank you for your time and consideration of our revised manuscript. We look forward to hearing from you.

Sincerely,

Hanh Van Nguyen

Faculty of Education, Hanoi University of Science and Technology, Hanoi, Vietnam

Email: hanh.nguyenvan@hust.edu.vn

Attachment Submitted filename: Response to Editor and Reviewers Final.pdf

# Editor report: Decision Letter 2

**DOI:** 10.1371/journal.pone.0359549.r005

**Authors:** Haiyang Chen

15 Sep 2026

<p>Perceived factors associated with self-reported AI-assisted academic cheating among undergraduate students: Evidence from ethical ambiguity, technological affordances, institutional gaps, academic pressure, and peer comparison

PONE-D-26-13247R2

Dear Dr. Van-Hanh,

We’re pleased to inform you that your manuscript has been judged scientifically suitable for publication and will be formally accepted for publication once it meets all outstanding technical requirements.

One small correction: your response letter says the gender–AICB association “was not attenuated” after adjusting for field of study. The coefficient drops from β = .183 to β = .142, and the manuscript correctly describes this as modest attenuation. Please fix that wording in the response letter for the editorial record. This does not affect the acceptance decision. No further analyses or review are needed. Please make sure the clean manuscript, not the tracked-changes version, is the version of record.

An invoice will be generated when your article is formally accepted. Please note, if your institution has a publishing partnership with PLOS and your article meets the relevant criteria, all or part of your publication costs will be covered. Please make sure your user information is up-to-date by logging into Editorial Manager at Editorial Manager® and clicking the ‘Update My Information' link at the top of the page. For questions related to billing, please contact billing support.

If your institution or institutions have a press office, please notify them about your upcoming paper to help maximize its impact. If they’ll be preparing press materials, please inform our press team as soon as possible -- no later than 48 hours after receiving the formal acceptance. Your manuscript will remain under strict press embargo until 2 pm Eastern Time on the date of publication. For more information, please contact onepress@plos.org.

Kind regards,

Haiyang Chen

Academic Editor

PLOS One

# Editor report: Acceptance letter

**DOI:** 10.1371/journal.pone.0359549.r006

**Authors:** Haiyang Chen

PONE-D-26-13247R2

PLOS One

Dear Dr. Van-Hanh,

I'm pleased to inform you that your manuscript has been deemed suitable for publication in PLOS One. Congratulations! Your manuscript is now being handed over to our production team.

At this stage, our production department will prepare your paper for publication. This includes ensuring the following:

* All references, tables, and figures are properly cited

* All relevant supporting information is included in the manuscript submission,

* There are no issues that prevent the paper from being properly typeset

You will receive further instructions from the production team, including instructions on how to review your proof when it is ready. Please keep in mind that we are working through a large volume of accepted articles, so please give us a few days to review your paper and let you know the next and final steps.

Lastly, if your institution or institutions have a press office, please let them know about your upcoming paper now to help maximize its impact. If they'll be preparing press materials, please inform our press team within the next 48 hours. Your manuscript will remain under strict press embargo until 2 pm Eastern Time on the date of publication. For more information, please contact onepress@plos.org.

You will receive an invoice from PLOS for your publication fee after your manuscript has reached the completed accept phase. If you receive an email requesting payment before acceptance or for any other service, this may be a phishing scheme. Learn how to identify phishing emails and protect your accounts at https://explore.plos.org/phishing.

If we can help with anything else, please email us at customercare@plos.org.

Thank you for submitting your work to PLOS One and supporting open access.

Kind regards,

PLOS One Editorial Office Staff

on behalf of

Dr. Haiyang Chen

Academic Editor

PLOS One
